# Replisome stall events have shaped the distribution of replication origins in the genomes of yeasts

**DOI:** 10.1093/nar/gkt728

**Published:** 2013-08-19

**Authors:** Timothy J. Newman, Mohammed A. Mamun, Conrad A. Nieduszynski, J. Julian Blow

**Affiliations:** ^1^College of Life Sciences, University of Dundee, Dundee, DD1 5EH, UK, ^2^School of Engineering, Physics and Mathematics, University of Dundee, Dundee, DD1 4HN, UK and ^3^Centre for Genetics and Genomics, University of Nottingham, Nottingham, NG7 2UH, UK

## Abstract

During S phase, the entire genome must be precisely duplicated, with no sections of DNA left unreplicated. Here, we develop a simple mathematical model to describe the probability of replication failing due to the irreversible stalling of replication forks. We show that the probability of complete genome replication is maximized if replication origins are evenly spaced, the largest inter-origin distances are minimized, and the end-most origins are positioned close to chromosome ends. We show that origin positions in the yeast *Saccharomyces cerevisiae* genome conform to all three predictions thereby maximizing the probability of complete replication if replication forks stall. Origin positions in four other yeasts—*Kluyveromyces lactis*, *Lachancea kluyveri*, *Lachancea waltii* and *Schizosaccharomyces pombe*—also conform to these predictions. Equating failure rates at chromosome ends with those in chromosome interiors gives a mean per nucleotide fork stall rate of ∼5 × 10^−8^, which is consistent with experimental estimates. Using this value in our theoretical predictions gives replication failure rates that are consistent with data from replication origin knockout experiments. Our theory also predicts that significantly larger genomes, such as those of mammals, will experience a much greater probability of replication failure genome-wide, and therefore will likely require additional compensatory mechanisms.

## INTRODUCTION

During S phase of the eukaryotic cell division cycle, the entire genome is precisely duplicated. Because of the large size of eukaryotic genomes, this is achieved by activating hundreds or thousands of replication forks initiated bidirectionally from replication origins (ROs) distributed at locations throughout the genome. To maintain genetic stability, it is critical that no segment of DNA is replicated more than once in a single cell cycle. This means that no replication forks should be initiated on a section of DNA that has already been replicated. Eukaryotic cells solve this problem by dividing the process of replication into two non-overlapping phases (1,2). From late mitosis until the end of G1, before DNA synthesis begins, cells license ROs for use by loading them with double hexamers of the MCM2-7 (minichromosome maintenance) proteins. During S phase, these MCM2-7 hexamers become activated to form the core of the replicative helicase that drives progression of replication forks along template DNA. Before entry into S phase, the machinery that licenses new ROs is inactivated. This prevents re-replication of DNA by ensuring that each RO can only activate a single bidirectional pair of replication forks. In effect, the presence of MCM2-7 on DNA marks the origin as not having been replicated in the current cell cycle.

It is therefore critical that a sufficient number of origins are licensed before cells enter S phase. This issue is made more pressing because it is known that replication forks can irreversibly stall, e.g. if they encounter damaged (chemically modified) DNA. At present, it is not clear why stalling becomes irreversible and whether this involves removal of replication fork proteins from the DNA (3). If two converging replication forks irreversibly stall, the cell will have a major problem in replicating the intervening DNA. Cells cannot license a new origin between the two stalled forks, as this would also allow the re-licensing of origins on replicated DNA, leading to re-replication.

**Figure 1.**
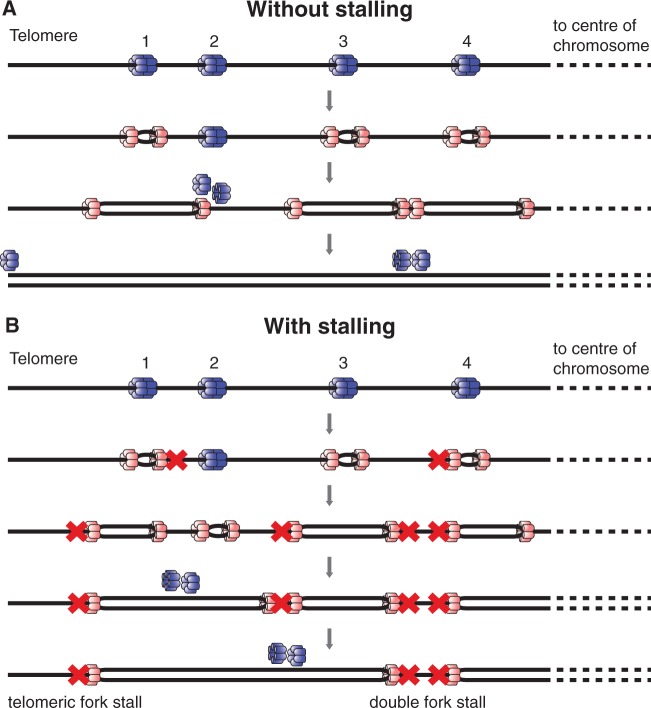
Cartoon of ROs near the end of a chromosome. DNA is denoted as a single black line, with a telomere (chromosome end) to the left. Before S phase entry, origins are licensed by binding a double hexamer of Mcm2-7 proteins (blue). As an origin fires, both Mcm2-7 single hexamers are converted into an active CMG helicase (pink). (**A**) Forks initiate at origins 1, 3 and 4. If an active fork passively replicates an inactive origin, the Mcm2-7 at the inactive origin is displaced making the origin dormant (origin 2) for that particular cell cycle. (**B**) In case of irreversible fork stalling (denoted by a red cross) otherwise dormant origins can be activated (origin 2) to ensure complete replication of the DNA. If both of the converging forks stall (‘double fork stall’) without a dormant origin existing between them (as occurs at forks converging between origins 3 and 4), replication of the intervening DNA is compromised. If the single fork heading towards the telomere (the fork move left from origin 1) irreversibly stalls and there is no telomere-distal origin, (‘telomeric fork stall’), then this single stall event can also compromise full replication of the genome.

Instead, cells protect themselves from the consequences of irreversible fork stalling by licensing many more origins than are normally used during S phase (4–7). When a replication fork encounters an inactive MCM2-7 double hexamer at a licensed origin, the inactive MCM2-7 double hexamer is displaced from the DNA, returning the origin to the unlicensed state. However, if two converging replication forks stall, ROs that would otherwise remain dormant can be activated between them to ensure that the genome is completely duplicated. In Figure 1A, origin 2 is passively replicated by a fork progressing rightwards from origin 1; in this case, origin 2 does not fire and so is ‘dormant’. In Figure 1B, the fork progressing rightwards from origin 1 stalls before reaching origin 2, which subsequently fires ensuring that all the DNA between origins 1 and 2 is replicated. Experimental work suggests that in most eukaryotes, there is a 3–10-fold excess of dormant origins over origins that actually fire (7–10). Origins are probably made dormant simply by virtue of being relatively inefficient so that they do not fire in the majority of cell cycles. However, the key requirement for complete genome duplication appears to be the number and distribution of licensed ROs, rather than the efficiency with which they are normally used (11). Replication can fail if two converging forks irreversibly stall, with no dormant origin between them (Figure 1B, ‘double fork stall’). The ends of linear chromosomes (telomeres) represent a special case, as they can only be replicated by forks coming from a single direction, from the body of the chromosome. Replication can fail at chromosome ends if a single replication fork stalls in telomeric or subtelomeric DNA, and there is no other licensed origin distal to the stalled fork (Figure 1B, ‘telomeric fork stall’).

In bacteria, replication imposes constraints on genome organization; genes tend to reside on the leading DNA strand with highly expressed and essential genes located close to the origin of replication (12). Bacterial chromosomes are typically circular, though defined fork termination sites mean that most of the genome can only be replicated by a single fork. Although considerable data has been obtained describing the location of ROs in eukaryotic cells (13,14), relatively little is known about the constraints that govern their number and distribution. In many eukaryotic cell types (most notably in animal cells), ROs are found at a large range of different genomic loci but are normally only used inefficiently. This means that in any given cell passing through S phase, most origins remain dormant and do not fire, but instead are passively replicated by forks initiated at neighbouring origins. At present, it is unclear the extent to which this reflects the inefficient licensing of ROs or the inefficient firing of licensed origins. In contrast, the budding yeast *Saccharomyces cerevisiae*, whose replication programme has been intensively studied, displays significantly more efficient origin use than is typically seen in eukaryotes (15–18). This makes *S. cerevisiae* a good model organism to study questions related to the number and distribution of ROs.

In this article, we construct a simple model of DNA RO distribution and use probability theory to quantify the degree to which replication fork stalling leads to incomplete replication of the genome. We then show that the numbers and distribution of origins in the *S. cerevisiae* genome conform to predictions made by our model, a conclusion supported by analysis of four other yeast species. In addition, our model allows an estimate of the per nucleotide fork stall rate and predicts that overabundance of ROs may not be sufficient to ensure robust replication in organisms with significantly larger genomes than *S. cerevisiae.*

## MATERIALS AND METHODS

### The model

We first constructed a simplified model of DNA replication. The process is summarized in Figure 1 and is based on the following assumptions and definitions:

- Replication forks can only originate at licensed ROs,
- Licensed origins are established at specific sites on the genome prior to any replication forks being activated,
- When an origin fires, replication forks are activated, travelling in opposite directions (bidirectionally) along the DNA; as this happens, the origin reverts to the unlicensed state,
- Licensed origins yet to fire are inactivated if they are visited by replication forks originating from another origin,
- Each replication fork has a constant independent probability *q* per nucleotide of irreversibly stalling (or otherwise failing),
- The average separation (in base pairs) between licensed origins is defined to be 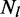,
- The total length of the genome is defined to be 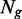,
- The median stalling distance of a replication fork is defined to be 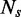,
- We assume the hierarchy: 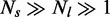,
- The DNA at the extreme ends of a chromosome that extends from the last RO (the ‘subtelomeric origin’) to the telomere represents a special case, as it can only be replicated by a single fork.
- We assume no upper time limit for replication of the entire genome.

### Probability of double stalls

We denote by *D* the region of DNA between two adjacent ROs and denote nucleotides in *D* by an integer variable *n*. Let the left RO be located at *n* = 0, and the right RO be located at *n* = *N*. The probability of a double stall in *D* is given by the following expression:
(A1)
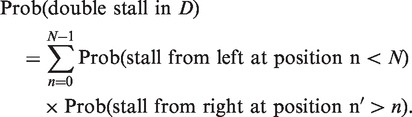



Now, if *q* is the mean per-nucleotide stall rate:
(A2)
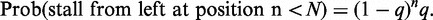

Similarly,
(A3)
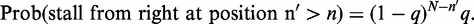

We need to sum Equation (A3) over all possible 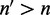 to give the total probability of a stall from the right (i.e. left-moving) RO that occurs at a site to the right of the stalled left RO located at 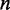. So,
(A4)
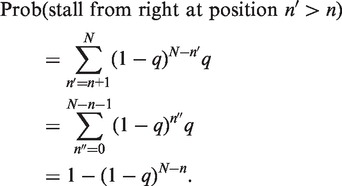



For clarity, we have defined a new summation variable 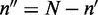 for the sum and used the following formula for summation of a geometric series:
(A5)
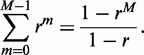



Inserting together Equations (A2) and (A4) we have
(A6)
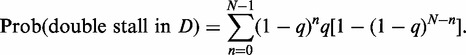



These sums are geometric series and hence can be explicitly evaluated using Equation (A5), and thus we get the simple exact result:
(A7)
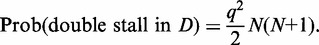



As the typical distance between licensed origins 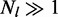, we can simplify this exact result to
(A8)
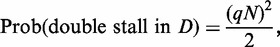

By the definition of 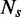 (the median stalling distance), we have
(A9)
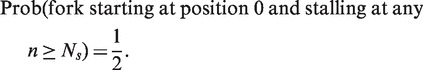



Let us denote this long-winded probability by 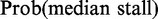. Now, according to Equation (A2), we have
(A10)
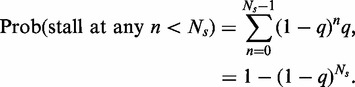



So,
(A11)
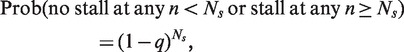

which means,
(A12)
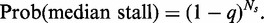



According to Equation (A9), we have an exact relationship between 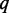 and 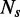:
(A13)
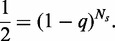



Now, taking natural logarithms, we have:
(A14)
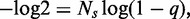

As 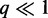, 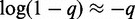, and thus we derive the following expression
(A15)
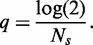



We can use Equation (A15) to write Equation (A8) purely in terms of 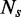 and we get
(A16)
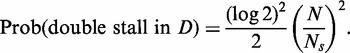

Defining the constant 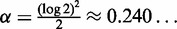 we have
(A17)
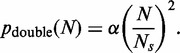

as given in Equation (1) in the main text.

### Spatial variation in ROs

We denote the separation between the neighbouring ROs labelled by 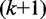 and 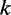 by 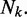 Now, associated with this pair of ROs is the probability of a double stall 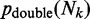, and we denote this by 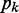, just for convenience. So, we have
(A18)
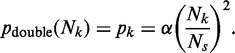



Now, we denote the probability of no double stall genome wide by 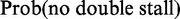, which is simply given by the following product of independent probabilities for no double stall in every possible region of separation between adjacent ROs:
(A19)
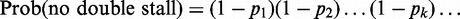



or,
(A20)
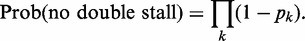



Using the fact that a product of factors can be rewritten as the exponential of a sum of logarithms of these factors, we can rewrite the above equation in the following form
(A21)
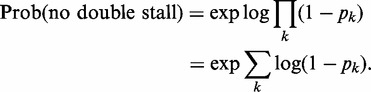



Now, as we have assumed that 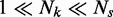 for all 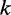, the value of 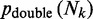 or 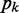, which is 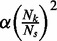, implies that 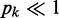. Thus, 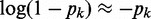 and Equation (A21) takes the following simpler form
(A22)
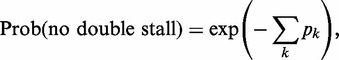

as given in Equation (2) in the main text.

We define an average of the independent quantities 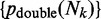 or 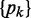, and their overall number. We denote the average by 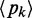. The overall number is the size of the genome divided by the average inter-RO distance (denoted by 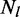 in the article), that is (approximately) 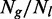. Then the law of large numbers provides us with the relation:
(A23)
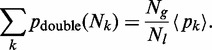

But, as we know 
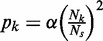
, we can directly relate 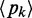 to the second moment of inter-RO distance 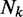 i.e.
(A24)
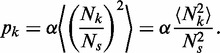

Now, using Equation (A24), we rewrite Equation (A23) as below
(A25)
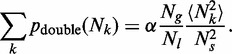



So, it is clear to write Equation (A22) as
(A26)
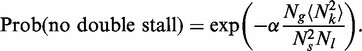



The second moment of a distribution is equal to the square of the mean plus the variance., denoting the variance in the inter-RO separation by 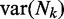, we have
(A27)
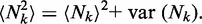

By definition, 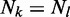, and so we combine Equations (A27) and (A26) to write Equation (A26) more explicitly in terms of variance:
(A28)
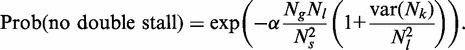

By replacing the variance with the standard deviation of the inter-RO distances 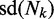, we have
(A29)
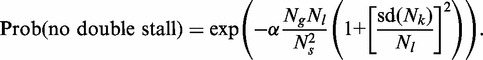

We denote the ratio of standard deviation to mean, 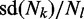, as *R* in our article and thus we have
(A30)
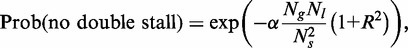

as given in Equation (3) in the main text.

Now,

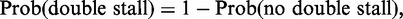

(A31)
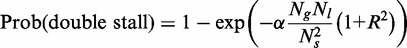



In the event that this probability is small, meaning 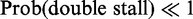, in which case the argument of the exponential must be small, and we have
(A32)
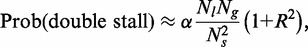

as given in Equation (4) in the main text.

### Error from the largest origin separation

We denote the largest gap between adjacent ROs in the given data set by 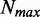. Now, from Equation (A18), we can directly write the double stall probability for the specific inter-RO separation denoted by 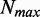, as following
(A33)
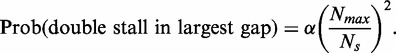

which together with Equation (A32) leads to Equation (5) in the main text.

### Errors at chromosome ends

According to Equation (A2), we write
(A34)
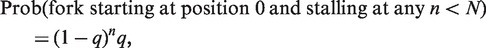

or probability of a single fork stall at any *n* within a specified region is simply
(A35)
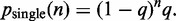



Thus, for a chromosome end, which has a length of 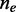 in bps, the single stall probability for a replicating fork can be given as
(A36)
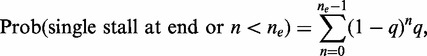

Using Equation (A5), we get
(A37)
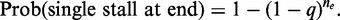

So, we can write
(A38)
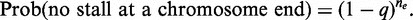



Let us consider the total number of chromosomes is *M*, and then the total length of all ends is 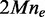, which is denoted by 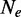 in our article. Now, the product of independent probabilities for no stall at every single end of all the chromosomes gives us
(A39)
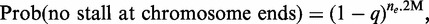

or
(A40)
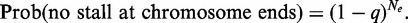



We can write this product in the form of exponential, using natural logarithms
(A41)
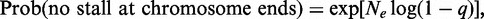



As *q* is small, 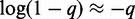 and we write
(A42)
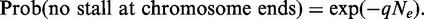



Now, we use Equation (A15) to rewrite Equation (A42) in terms of 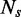

(A43)
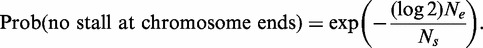

as given in Equation (6) in the main text. So, it is now straightforward to write,
(A44)
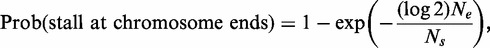

As 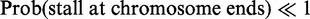, the argument of the exponential must be small, and we have
(A45)
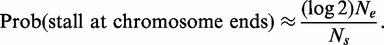

as given in Equation (7) in the main text.

Now from the arguments given in the article, we write
(A46)
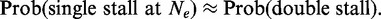

which means
(A47)
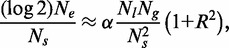

as given in Equation (8) in the main text.

Simply by considering 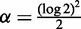 and slightly rearranging above expression, we have
(A48)
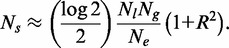

as given in Equation (9) in the main text.

### Data concerning origin distribution in yeast species

We selected *S. cerevisiae* RO locations based on the data at OriDB (19) using the following criteria:

1. All sites that have been experimentally confirmed by an Autonomously Replicating Sequence (ARS) assay (410 sites);
2. Additional sites that were identified in two independent high-resolution chromatin-immunoprecipitation studies of origin licensing proteins (18,20) (52 sites);
3. Telomeric origins that are predicted from sequence conservation with confirmed telomeric origins (23 sites);
4. We removed proposed origin sites that we previously experimentally showed to be false positives (21) (4 sites).

The resulting list contains 482 RO sites which are listed in Supplementary Data Set 1. This data set contains only a single copy of rDNA (9.1 kb in size and containing a single RO), which is duplicated ∼100 times in the genome (22).

We have taken the RO data for four other *Saccharomyces* species from previously published data sets consisting genome-wide RO positions in *Kluyveromyces lactis* (23), *Lachancea waltii* (24), *Lachancea kluyveri* (25) and *Schizosaccharomyces pombe* (26). Though these data sets do not have the accuracy of the *S. cerevisiae* data particularly in regard to the telomeric origins, they are strong enough to give analytical support to the origin distribution profile in *S. cerevisiae*. Genome and chromosome size information was obtained from the following sources: *K. lactis* (27), *L. waltii* (28), *L. kluyveri* (29) and *S. pombe* (30).

### Estimation of spontaneous stall rate in human cells

We estimate the spontaneous median stalling distance 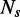 using DNA fibre data from reference (31) concerning MRC5 cells, a primary human cell line. The key data derive from analysis of DNA molecules labelled with 2 successive nucleotides: a 20 min pulse of BrdU directly followed by a 30 min pulse of biotin-11-dUTP. ‘Type 4’ structures consist of a BrdU track that is adjacent to but not contiguous with an isolated biotin track, and they must result from a fork stall. Reference (31) showed that in MRC5 cells 0.5% of all replication tracks showed a Type 4 pattern. The stall can have occurred either at the end of the BrdU track or in the intervening DNA between the BrdU and biotin labels before the BrdU labelling period. Further fibre analysis in (31) shows that tracks of ∼25 kb are normally labelled during the 20 min BrdU pulse, and that the average origin-to-origin separation is ∼72 kb. Consideration of the possible types of labelled structures (Supplementary Figure S1A) suggests that roughly one-third of all Type 4 structures would be caused by a fork stall that occurred after the pulse started. We therefore estimate that ∼0.16% (0.5%÷3) of all replication tracks labelled over 25 kb end in a stall. This represents a per nucleotide stall rate *q* of ∼6 × 10**^−^**^8^ (0.0016÷25 000). From Equation A15, this gives a median stall distance 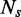 of ∼10 Mb, which should be considered only a rough estimate. A similar approximation is obtained using stall estimates derived from HeLa cells (31).

## RESULTS

### Probability of double stalls

To determine the effect of origin distribution on the probability of the genome being successfully duplicated, we developed a mathematical model of genome duplication (see ‘Materials and Methods’ section). One important assumption we make is that there is no upper time limit for replication of the entire genome. This is biologically plausible, as, for many cell types, DNA replication checkpoints activated when replication forks stall can extend the length of time available for S phase by delaying progression into mitosis. This model allows us to address the question of whether the entire genome can be fully replicated even if forks irreversibly stall, given sufficient time for all available origins to fire and for forks to progress along template DNA.

Our theory should be considered as complementary to previous theoretical work in which the number and distribution of origins have been considered in the light of optimizing error-free replication within a certain period (32–34). Timing is a major issue for cells such as early embryos that must license and then fire ROs within a fixed short period (35–37). Our theory is instead more likely to apply to cells freed from the constraint of rapid turnover (e.g. single-celled organisms or somatic cells in homeostatic tissues in adult metazoans) where ample time is available to fire dormant origins in response to replication fork stalls. A previous study (38) has used a numerical analysis of probability density equations to examine the effect of DNA damage on the completion of DNA replication where fork stalling is a function of exogenous DNA damage. However, because it assumed that the density of dormant origins is high, this article does not address the type of replication failure we are considering here.

Consider a region of the genome, denoted by the symbol *D*, which represents all the DNA between two adjacent ROs *N* base pairs apart (e.g. between ROs 1 and 2 in Figure 1). There is a certain small probability that a fork may stall irreversibly at each base pair that is replicated. The only way for *D* to contain unreplicated DNA after all origins have either fired or been inactivated (as a consequence of passive replication by a fork from another origin) is for two replication forks to have entered *D*, one from the left and the other from the right, and for both forks to have stalled before meeting within *D*, an event we refer to as a ‘double fork stall’. It is irrelevant for our purposes whether these forks originated from the origins located at either end of *D*, or whether they originated outside of *D* and entered by inactivating the licensed origins bounding *D*. Those are time-dependent details, while we ask here statistical questions concerning the final state of the DNA, assuming no time constraint on replication. A double fork stall within *D* is highly unlikely, as the average distance a fork will travel before stalling (the median stalling distance 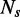) is much larger than the typical inter-origin separation. Using elementary probability theory (see ‘Materials and Methods’ section), we find that the probability of a double fork stall within *D* is:
(1)
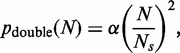

where 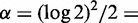 0.240 … .

### Spatial variation in RO spacing

We can use the model to examine the probability of double fork stalls occurring anywhere throughout the genome. We will leave aside for the time being events occurring at the ends of chromosomes where DNA can only be replicated by a single fork coming from the body of the chromosome. Although the stall rate may vary at different locations in the genome, the scale over which this varies is likely to be very much smaller than the median stalling distance 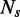, and therefore will not significantly affect our analysis. Chromosome fragile sites, which were once thought to be large chromosome domains where forks have an increased probability of stalling, instead appear to be regions containing a paucity of active ROs (39), which Equation 1 shows will be places where the probability of double stall events is high. First, we calculate the probability of no errors occurring through double stalls, genome-wide, for a given (fixed) initial distribution of origins. Let the index *k* label the positions of the licensed origins along the entire genome (as in Figure 1). The distance (number of base pairs) between any two adjacent origins must be much larger than 1, as the footprint of a single MCM2-7 double hexamer covers ∼70 bp (40,41). We denote the separation between the neighbouring origins labelled by 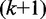 and 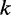 by 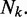 Now, associated with each pair of origins is the probability of a double stall 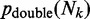. Application of probability theory (see ‘Materials and Methods’ section) provides us with an expression for the probability, genome-wide, of no double stalls occurring:
(2)
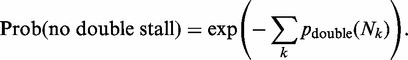



We assume that the separations between adjacent origins are distributed in a statistical sense, i.e. not being strictly determined by some ordered pattern. This does not necessarily imply complete randomness. For example, there may be strong spatial correlations between these distances. All we require is to define an average separation between adjacent origins and the existence of an associated standard deviation (i.e. we assume the distribution has no power law tail), both of which can be directly measured from RO data sets. Using the law of large numbers (see ‘Materials and Methods’ section) and Equation (1), we can re-express Equation (2) as follows:
(3)
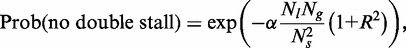

where 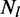 is the mean separation between ROs, 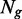 is the size of the genome and *R* is the standard deviation of separations divided by the mean. It is clear from this expression that the probability of no double stalls is maximized by setting 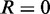. In other words, any degree of spatial variation of origin separations will serve to ‘increase’ the probability of a genome-wide replication error.

**Figure 2.**
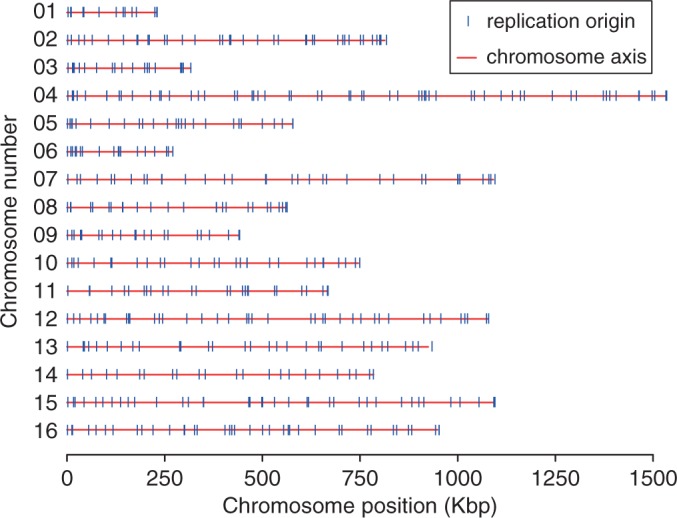
Positions of the 482 ROs in all 16 *S. cerevisiae* chromosomes as described in the data set used in this article. Each chromosome is denoted by a red line, with origins indicated by a vertical blue line.

**Figure 3.**
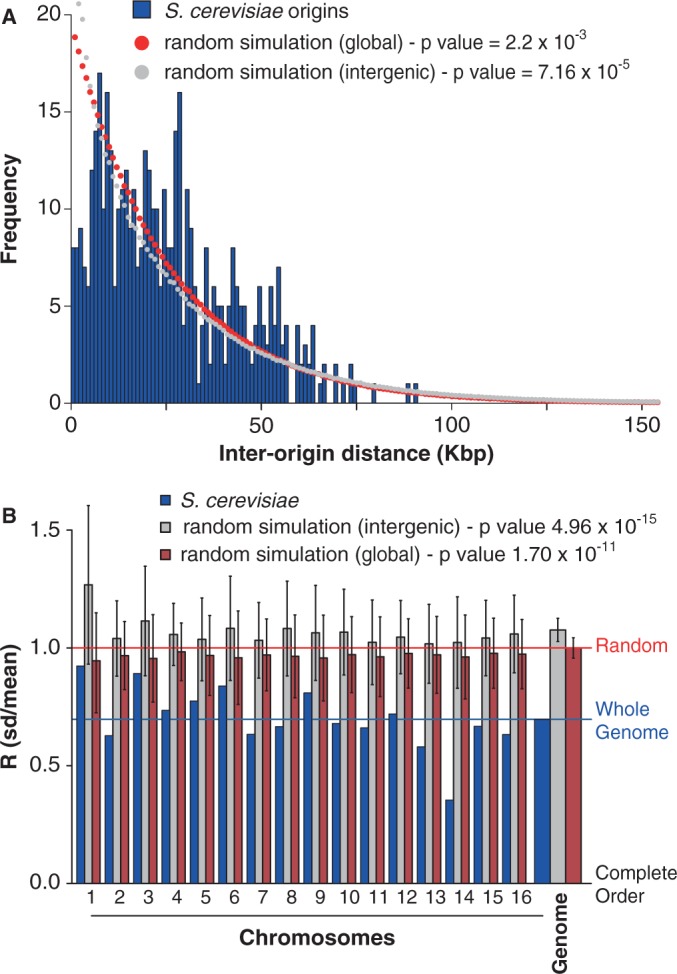
Inter-origin spacings in the *S. cerevisiae* genome. (**A**) Inter-origin spacings in *S. cerevisiae* were calculated and assigned to different 1 kb bins. The frequency of origins in each bin is shown. Red dots: mean origin separation in a computer simulation where the same number of origins were placed at random on the whole *S. cerevisiae* genome. Grey dots: mean origin separation in a computer simulation where the same number of origins were placed at random only in the intergenic regions of the *S. cerevisiae* genome. (**B**) *R*, the ratio of standard deviation divided by mean of the separations between RO positions was calculated for each *S. cerevisiae* chromosome and for the *S. cerevisiae* genome as whole (blue bars). *R* was also obtained by computer simulation for an equal number of origins positioned randomly, either globally throughout the genome (red bars) or in intergenic regions only (grey bars). Error bars show the standard deviation. A value of 0 for *R* represents completely regular origin spacing, whereas a value of 1 would represent random origin spacing on an infinitely long chromosome. The horizontal lines show the *R* value for the whole genome from the actual data set (blue) and from numerical simulation of randomly placed origins (red). *R* values were calculated from 10 000 iterations in the simulation.

If the avoidance of double fork stalls is an important factor in the positioning of ROs, Equation (3) suggests that they should be more regularly spaced than would be expected by chance. We therefore examined origin distribution in the yeast *S. cerevisiae*, where ROs have been mapped genome wide (Figure 2). We calculated the distances between adjacent origins for each of the 16 yeast chromosomes, considering the middle point of each ARS element in the given data set as the origin of replication (Figure 3A). For comparison, we performed a computer simulation where the same number of origins was placed on the *S. cerevisiae* genome at random (Figure 3A, red dots). The *in vivo* origin distribution is clearly more uniform than the random distribution, with fewer very small and very large inter-origin separations, with the difference giving a mean *P*-value (Kolmogorov–Smirnov test) of 2.22 × 10**^−^**^3^.

We then calculated *R*, the ratio of the standard deviation of origin separations divided by the mean, for each of the 16 chromosomes. Any deviation away from periodic spatial ordering of origins would give a value of *R* greater than zero. Complete randomness in the positions of origins provides an upper bound for *R*. There is a subtlety that this upper bound depends on the number of origins considered (or, equivalently, for a fixed mean separation, on the length of DNA considered). For origins randomly placed on an infinite strand of DNA, 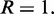 For finite strands, complete randomness yields a value of *R* < 1, and this must be used to correctly calibrate whether empirical data of origin separations reflect some degree of order or randomness (Supplementary Figure S2).

We therefore calculated *R* for each individual *S. cerevisiae* chromosome and compared the values with the value expected if the same number of origins had been randomly distributed along the chromosome (Figure 3B). All 16 chromosomes had an *R* value less than given by a random distribution. When all inter-origin distances are considered together, they give an *R* value of 0.697, compared with a value of 0.999 ± 0.046 for equivalent random distributions (Figure 3B). This means that the spatial distribution of origins in *S. cerevisiae* chromosomes is significantly below complete randomness, with the difference giving a *P* value of 1.70 × 10**^−^**^11^ (using a normal distribution, as shown in Supplementary Figure S3). The value of *R* changes very little if origins are randomly removed from or added to the data set, showing that it is robust to the presence of false positives or false negatives in the data set (Supplementary Figure S4). This suggests that minimizing the probability of double fork stalls has been an important influence on the positioning of ROs along the budding yeast genome. However, the inter-origin spacing has significantly more variation than complete order, and this presumably reflects an evolutionary trade-off between minimizing global error rates and the difficulty in creating perfect ordering of origins in a living cell.

It is known that in *S. cerevisiae* origin efficiency declines if transcription is driven through the origin (42,43). We therefore investigated whether the low values of *R* seen across the *S. cerevisiae* genome could be a consequence of origins being preferentially located in intergenic regions. We performed a simulation of origin position on the genome where origins were placed at random either throughout the genome (‘global’ simulation, Figure 3A and B) or only within intergenic regions (‘intergenic’ simulation, Figure 3A and B). In random simulations, restricting origins to intergenic regions increased the value of *R* over the genome by ∼8%. These data strengthen our observation that the *R* value of origin distribution is much lower than would be expected by chance.

To determine whether the regular spacing of origins is conserved throughout evolution, we analysed origin distribution in three other related yeasts *K. lactis* (Supplementary Figure S5A and B), *L. kluyveri* (Supplementary Figure S6A and B) and *L. waltii* (Supplementary Figure S7A and B). The ROs in 21 of the 22 chromosomes of these three organisms are significantly more evenly spaced than expected by chance. The *R* values for genome-wide origin distribution, shown in Table 1, are 0.55 (*K. lactis*; Supplementary Figure S5C), 0.46 (*L. kluyveri*; Supplementary Figure S6C) and 0.58 (*L. waltii*; Supplementary Figure S7C). The similar *R*-values in all of these yeast species are unlikely to be due simply to origin position being maintained over evolutionary time, as comparison of origins between *S. cerevisiae* and *K. lactis* (23) and between *S. cerevisiae* and *L. waltii* (24) showed that few origins have maintained a conserved location between the pairs of species. Instead, our results suggest that there is a strong evolutionary pressure to regularly space ROs in all four of these organisms. RO distribution in the distantly related fission yeast *S. pombe* was also non-random (Supplementary Figure S8), with an *R*-value for origin distribution of 0.86. The lower degree of origin spacing in *S. pombe* may reflect a different organization of ROs, which are defined by a much looser DNA sequence consensus (44,45) where most origins initiate replication in only a small proportion of cell cycles (46). In this sense, origin distribution in *S. pombe* more closely resembles what is seen in metazoan cells. The three yeasts with the largest mean inter-origin distances—*K. lactis*, *L. kluyveri* and *L. waltii*—also have the smallest *R* values: this may simply reflect that origin identification in these organisms has not been done at the depth of the other two organisms, but it is what would be expected if evolution is maintaining a certain tolerated value for the probability of double fork stalls, and therefore in these organisms, an increase in the mean distance between origins has been compensated for by making the origins more evenly spaced.

**Table 1.** Summary of the genome organization and RO distribution in five different yeasts

Species	*S. cerevisiae*	*K. lactis*	*L. kluyveri*	*L. waltii*	*S. pombe*
Genome size (Mb)	12.07 (13.0^a^)	10.7	11.3	10.2	12.6
No. of chromosomes	16	6	8	8	3
No. of origins	482	148	252	194	460
Origin separation (mean ± s.d.) (kb)	26 ± 18	71 ± 39	44 ± 21	52 ± 30	27 ± 23
*R*-value	0.70 (0.76^a^)	0.55	0.46	0.58	0.86
Simulated random *R*-value	0.999 ± 0.046 (1.077 ± 0.049^b^)	0.993 ± 0.079	0.996 ± 0.061	0.995 ± 0.071	0.998 ± 0.046
*P*-value for non-randomness of *R*^c^	3.93 × 10^−12^	1.09 × 10^−8^	3.56 × 10^−18^	1.28 × 10^−09^	1.45 × 10^−3^
Max. origin separation (kb)	90	219	102	203	116
Expected max. origin separation (kb)	169 ± 31 (182 ± 34^b^)	399 ± 85	273 ± 53	307 ± 64	183 ± 34

*R* refers to the ratio of the standard deviation to the mean of the origin separations.

^a^Considering 100 repeats of 9.1 Kb rDNA sequence in chromosome 12.

^b^Random placement of origins restricted to intergenic regions only.

^c^Using a Gaussian fit.

### Genome-wide replication failure rate and parameter bounds

We can use Equation (3) to provide a bound on the probability of one or more double stall errors in genome-wide replication. The probability of one or more double stall events is simply given by 1 minus the probability of no double stalls. In the event that this probability is small, as shown in ‘Materials and Methods’ section, it is straightforward to use Equation (3) to show:
(4)
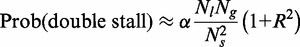



This global error rate is proportional to 1 + *R*^2^, which differs only by a factor of ∼2 between complete order (*R* = 0) and complete disorder (*R* ≈ 1), so although not insignificant, there will probably not be a hard selective pressure on origin distributions. The measured value of 0.70 gives 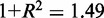, almost exactly mid-way between the two extreme values of 1 and 2.

Intriguingly, the three fundamental scales 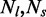 and 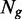 appear in the quotient 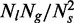. Details of the relative sizes of these scales are crucial in determining the order of magnitude of the error. Even though we have a strict hierarchy 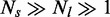, we cannot infer anything about the size of this quotient without first estimating the relative size of the genome with respect to the median stalling distance. We will return to this point in the ‘Discussion’ section, when comparing yeast and mammalian genomes.

In *S. cerevisiae*, we know the value of 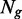 for unique sequence DNA as ∼12.1 Mb (13.0 Mb if repetitive DNA is also considered; see Table 1), and from our data set, we have the value of 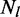 (the average distance between origins) as 25 868 and 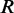 as 0.70. However, reliable estimates for the median stall distance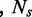, are hard to obtain because by necessity it has to be significantly larger than the average replicon size. DNA fibre studies in human cells provide data suggesting a mean stall distance of ∼10 Mb in the absence of checkpoint or replication inhibitors (mean per nucleotide stall rate of ∼6 × 10**^−^**^8^) (31, see ‘Materials and Methods’ section for derivation). Using Equation (4) with a median stall distance of 10 Mb gives a value of 0.11% for the probability of a double fork stall in *S. cerevisiae*. Studies on chromosome segregation in *S. cerevisiae* have shown that individual chromosomes missegregate in ∼2 × 10**^−^**^5^ of all cell divisions (47,48). Therefore, any one of the 16 chromosomes would be expected to missegregate in ∼0.032% of all mitoses (16 × 2 × 10^−^^5^), which is within a factor of three of our predicted double stall rate. This order of magnitude equivalence might be expected because both DNA replication errors and chromosome segregation errors contribute to genome instability. Using Equation (4) with the same median stall distance of 10 Mb predicts that the probability of a double fork stall in the other yeasts is similar: 0.24 (*K. lactis*), 0.14 (*L. kluyveri*), 0.17 (*L. waltii*) and 0.14% (*S. pombe*). The similarity between all these numbers suggests that evolution has maintained a certain tolerated value for the probability of double fork stalls that is of a similar magnitude to the tolerated chromosome missegregation rate.

### The largest inter-origin separation

**Figure 4.**
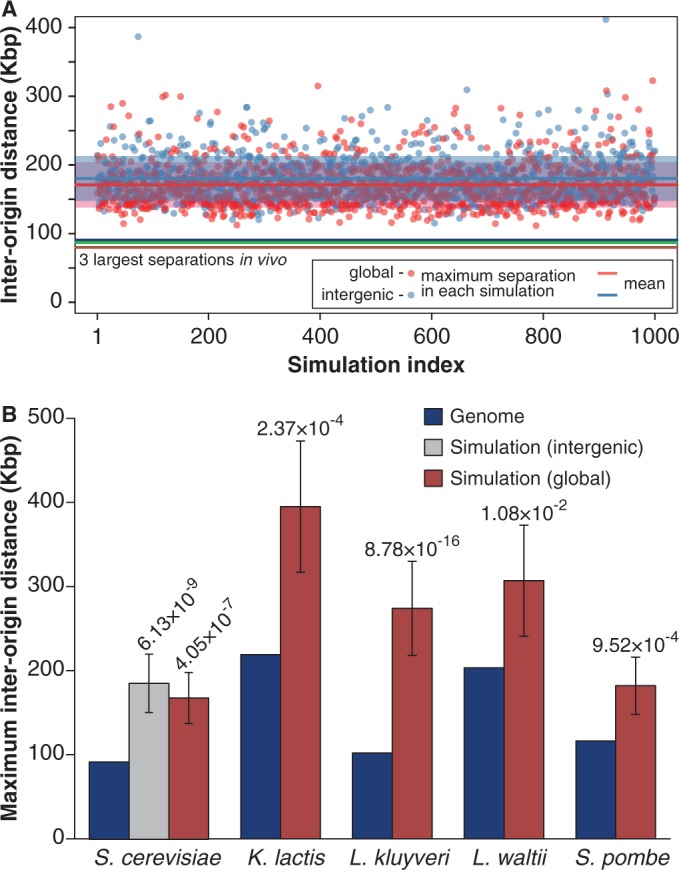
Analysis of the maximum inter-origin distances. (**A**) The three largest inter-origin distances in the *S. cerevisiae* genome (90.1, 88.5 and 79.3 kb) are shown as horizontal lines (blue, green, brown). A computer simulation was performed for an equal number of randomly positioned origins. Red dots are the maximum origin separation in each of the simulations when origins were placed randomly throughout genome (1000 shown). The mean of these simulated values is 169 kb (red line) with a standard deviation of 31 kb (shaded box around the red line), calculated from 10 000 simulations. Blue dots are the maximum origin separation in each of the simulations when origins were randomly placed only in intergenic regions (1000 shown). The mean of these intergenic simulation values is 182 kb (light blue line) with a standard deviation of 34 kb (shaded box around the light blue line), calculated from 10 000 simulations. (**B**) Blue bars show the largest origin separations in *S. cerevisiae*, *K. lactis*, *L. kluyveri*, *L. waltii* and *S. pombe*. A computer simulation was performed for an equal number of randomly positioned origins on genomes of the appropriate size (red bars); for *S. cerevisiae*, a similar simulation was performed with origins being restricted to intergenic regions (grey bar). For each simulation condition, the mean and standard deviation of the maximum separations derived from 10 000 simulations is shown. Figures above the bars are *P*-values of the difference between the real and random values estimated using a Gumbel fit.

Equation (1) shows that the probability of a double stall occurring between two adjacent origins is proportional to the square of the distance between them. This means that double fork stalls are proportionally more likely to occur between the most widely spaced origins. Even if the spacing of origins can be described by a narrow statistical distribution, given the large number of origins in a genome, there is a possibility for occasional large separations to arise, which may lie far beyond the standard deviation, and which may significantly increase the chances of double fork stalls. The three largest gaps between adjacent origins in the *S. cerevisiae* genome are 90.1, 88.5 and 79.3 kb. As a comparison, we performed simulations where origins were randomly distributed across the entire genome and for each simulation, the largest gap between the adjacent origins was determined (Figure 4A). The average maximum gap in the simulations was 169 ± 31 kb, and the minimum value obtained in the simulations was 116 kb. These simulated values are considerably larger than 90 kb observed in the *S. cerevisiae* genome, with the difference giving a *P*-value of 4.05 × 10**^−^**^7^ (using a Gumbel extreme-value distribution, as shown in Supplementary Figure S3). If the randomly positioned origins were restricted to intergenic regions only, the maximum gap increased to 182 ± 34 kb with a *P*-value of 6.13 × 10**^−^**^9^ for the difference between real and simulated data (Figure 4B). This provides further evidence that the RO distribution in yeast has been determined at least in part to minimize the consequences of irreversible fork stalling. When maximum inter-origin separations were analysed in the four other yeast species (*K. lactis*, *L.kluvyveri, L. waltii* and *S. pombe*), they were again seen to be significantly smaller than would be expected by chance (Figure 4B, Table 1 and Supplementary Figures S5D, S6D, S7D and S8D). These results strongly suggest that limiting the maximum separation between ROs is an important evolutionarily conserved feature of origin distribution.

Because the largest inter-origin spacing is the spacing in which a double fork stall is most likely to occur, it is worth providing a specific consideration of the probability of a double fork stall event occurring in this large region. We denote by 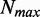 the size of the largest inter-origin spacing in the entire genome. We can use Equation (1) in conjunction with Equation (4) to approximate the proportion of double stalls in the largest inter-origin region (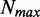) relative to all expected double stalls genome-wide:
(5)
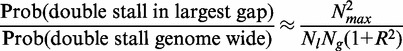



Substituting the known *S. cerevisiae* values of 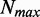, 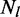, 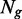 and *R* into this equation shows that 1.8% of all double fork stalls would be expected to occur in the largest inter-origin gap of 90 kb, which represents 0.7% of the total genome. In contrast, had origins been placed at random on the genome, the largest inter-origin gap would have an expected value of 169 kb and 4.6% of all double fork stalls would occur in this region.

Experimental work by Newlon and colleagues has investigated the consequences of deleting ROs in *S. cerevisiae* to create large origin-less regions (49). Deleting the five efficient origins on chromosome III between ARS304 and ARS313 creates an origin-less region of 160 kb (49, construct 5ORIΔ), close to the average maximum gap in the simulations of randomly positioned origins shown in Figure 4A. The loss rate of the 5ORIΔ chromosome was ∼9 × 10**^−^**^5^ per cell cycle, significantly larger than the loss rate of ∼3 × 10**^−^**^5^ shown by a comparable test chromosome, 0ORIΔ-ΔR without any deleted origins. This implies that the existence of the origin-less region creates an additional error rate of ∼6 × 10**^−^**^5^ per cell cycle. Applying Equation (1) with a median stall distance of 10 Mb and a 160 kb inter-origin spacing gives a value of 6.1 × 10**^−^**^5^ for the probability of a double fork stall rate occurring in this origin-less region, in excellent agreement with these observed results. This provides strong support to our theory and also suggests that 10 Mb is a reasonable approximation for the spontaneous median stall distance in *S. cerevisiae*.

### Fork stalling at chromosome ends

**Figure 5.**
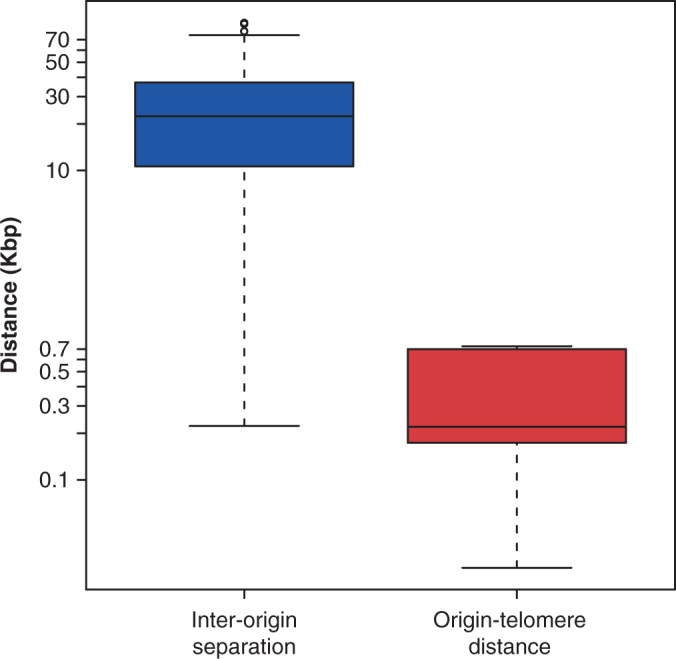
Comparison of inter-origin and end-origin distances. All the inter-origin distances and all 32 distances between the most end proximal RO to the telomere in *S. cerevisiae* are presented in a box plot. Note logarithmic *y*-axis.

The genome of eukaryotic cells is arranged on linear chromosomes. Consequently, if a single fork stalls in the telomeric regions of the chromosomes that lie beyond the last RO, there are no other forks that can replicate the telomeric DNA (Figure 1B, ‘telomeric fork stall’). It is notable that for each of the 16 *S. cerevisiae* telomeres, the closest origin is on average only 404 ± 273 bp away from the chromosome end. This is much smaller than the average distance between ROs in the chromosome body, which is 25 868 bp (Figure 5). Indeed, the maximum distance from a chromosome end to the first RO is 730 bp across all 32 chromosome ends. This again provides strong evidence that the distribution of ROs on the *S. cerevisiae* genome has been arranged to ensure complete replication in the face of replication fork stalling. Because of the repetitive nature of subtelomeric DNA, high-quality RO data are not available in this region for the other yeasts.

Given a cell with *M* chromosomes, we denote by 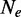 the sum length of these 2*M* end regions distal to the last origin. As shown in ‘Materials and Methods’ section, the probability of no fork stalls in the end regions of all *M* chromosomes is given by:
(6)
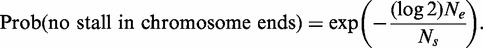

The probability of one or more such error events will be 1 minus the expression above, and if such errors are rare, then, as shown in ‘Materials and Methods’ section, one simply has:
(7)
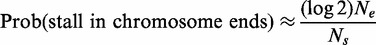



To keep a balance in the minimum error rate during replication, rates of replication failure at chromosome ends due to telomeric stalls should be similar to rates of replication failure in the body of chromosomes due to double fork stalls. Thus from Equations (4) and (7), we have:
(8)
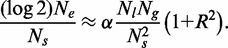



Manipulation of Equation (8) allows us to derive an approximation for 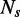:
(9)
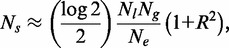

In *S. cerevisiae*, 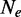 is 12 696 bp. Using this with the empirical values of 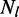, 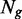 and *R* as discussed in the previous subsection, together with Equation (9) gives 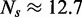 Mb (per nucleotide stall rate of 5.4 × 10**^−^**^8^), which is remarkably similar to the estimated stall distance of 10 Mb observed in mammalian cells (31). The coincidence of these different estimates of the stall distance gives us confidence that this value is approximately correct and that it has had an important influence on determining the spacing of origins in the *S. cerevisiae* genome. We also note the odd coincidence that this value is remarkably close to the size of the yeast genome.

Newlon and colleagues have also compared the effect of having a 160 kb origin-less region in the body of the chromosome (49, construct 5ORIΔ) with the effect of having the same origin-less region at the end of the chromosome (49, construct 5ORIΔ-ΔR). Truncating the chromosome so that the origin-less region is at the end of the chromosome increased the loss rate >20-fold (from a loss rate of ∼9 × 10**^−^**^5^ per cell cycle to ∼210 × 10**^−^**^5^), consistent with our hypothesis that the unidirectional nature of telomeric replication increases the chance of replication failure. The magnitude of this effect is somewhat smaller than our theory would predict: with an 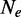 of 160 kb and 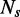 of 10 Mb, Equation (7) gives a predicted loss rate of ∼1000 × 10**^−^**^5^ per cell cycle, a factor of five times larger than that observed; this may imply that additional mechanisms exist at telomeric ends to allow complete replication.

## DISCUSSION

### Consequences of fork stalling are minimized by appropriate origin abundance and spacing

We have presented a theoretical analysis that determines the probability that genome replication fails due to irreversible stalling of replication forks. If a replication fork irreversibly stalls within the body of chromosomes, the DNA distal to it can still be replicated by a converging replication fork initiated at an adjacent origin. However, if this converging fork also suffers an irreversible stall—a double fork stall—there will be major problems to replicate the intervening DNA. We provide an equation Equation (4), which relates the genome size, the natural fork stall rate and the statistics of origin distribution to estimate the probability of a double fork stall occurring. This shows that the probability of double fork stalling will be minimized if ROs are regularly spaced. It will also be minimized if large inter-origin distances are avoided.

To test these predictions against biological data, we analysed origin distribution in the genomes of five different yeasts, including *S. cerevisiae*. The results clearly show that origin spacing is much more regular than would be expected by chance. In addition, we show that the largest gap between adjacent origins is significantly smaller than would be expected by chance. Experimental creation of a large origin-less region within a chromosome in *S. cerevisiae* increased the chromosome loss rate exactly in line with our predictions (46). These observations are consistent with the idea that the distribution of ROs within yeast chromosomes has been influenced by selection to ensure complete replication in the face of double fork stalling. Evenly spaced origins may also help to shorten the total length of S phase, but this effect is likely to be small compared with the effect of different initiation times, which create the extended replication timing programmes observed in *S. cerevisiae* and *S. pombe* (16,46,50). Previous computer modelling has shown that protection against double-fork stalling depends on the number of origins licensed, irrespective of whether these origins are efficient or whether they normally remain dormant (11). If origin efficiency falls below a critical value, the time taken to replicate a DNA segment is decreased by clustering origins together, rather than by spacing them evenly (34). We therefore conclude that the origin distribution we observe in yeasts is largely driven by the effect of fork stalling.

Replication of the extreme ends of chromosomes is particularly susceptible to the consequences of fork stalling because there is no possibility of replication being rescued by a converging fork. Our theoretical analysis suggests that the most terminal origin at each telomere should be much closer to the chromosome end than the average spacing between ROs within chromosomes. In accordance with this prediction, we show that in the *S. cerevisiae* genome, the average distance from the telomere proximal origins and the chromosome end is ∼50 times closer than the average spacing between origins. Experimental creation of a large origin-less region at the end of a chromosome in *S. cerevisiae* increased the chromosome loss rate broadly in line with our predictions (46). Mapping ROs near telomeres is technically challenging, and reliable data from other yeasts are not available. However, we note that in human cells, replication often initiates within the subtelomere and may even initiate within telomere repeats (51). It therefore appears likely that origins are positioned close to chromosome ends in many organisms, and that this provides an important mechanism for minimizing the consequences of fork stalling.

Our model allows us to predict the probability that replication fork stalling would potentially leave segments of the genome unreplicated. Using an estimate for the mean stall distance from human cells, the model predicts that *S. cerevisiae* would experience a double fork stall in ∼0.11% of S phases. This is a plausible value as it is only slightly higher than the total spontaneous chromosome loss rate, which also contributes to genetic instability. To minimize the probability of genome replication being incomplete due to replication fork stalls, the probability of fork stalling at chromosome ends should be similar to the probability of a double fork stall within the chromosome body. This equivalence allowed us to estimate the median stall distance in *S. cerevisiae* as ∼12.7 Mb, remarkably similar to the mean value of ∼10 Mb obtained in human cells (31). Chromosome loss rates derived from the artificial creation of origin-less regions (46) are also consistent with a median stall rate of ∼10 Mb.

These three observations about origin spacing in *S. cerevisiae* (regularity of origin spacing, small maximum inter-origin gaps and the position of telomeric origins), their conservation in other yeasts and their compatibility with plausible estimates of stall rates strongly support the idea that the positioning of ROs has been strongly influenced by the need to minimize the deleterious consequences of replication fork stalling. However, there are likely to be other factors that also influence origin position. First, other activities apart from DNA replication take place on the genome, and some of these may clash with DNA replication. For example, it seems likely that origin efficiency declines if transcription is driven through the origin (42,43). Second, the spontaneous stall rate is likely to vary across the genome and is likely to be strongly influenced by DNA sequence and the presence of proteins tightly bound to the DNA. Third, genomes are typically replicated according to a strict timing programme, which may impose constraints on the position of ROs. Fourth, both mutation rate and mutational asymmetry are influenced by the location and activation times of ROs (52,53). For example, mutation rates correlate with replication time in yeasts, flies and humans (54–56). Despite all the competing considerations, our results suggest minimizing the consequences of fork stalls still appears to be a major consideration.

### Effect of genome size on the probability of double fork stalls

Our equation for the probability that a double fork stall occurs has as its primary component the quotient 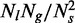. For budding yeast, we have provided evidence that 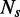, the natural fork stall rate, is of the same order of magnitude as 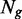, the genome size. As 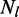, the average distance between licensed origins, is much smaller than 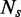, this means that under unstressed conditions, double fork stalls will be rare in the *S. cerevisiae* genome.

Many eukaryotes, in particular vertebrates, have genomes significantly larger than those of yeasts. In particular, the diploid human genome is ∼6000 Mb in size, 500 times the size of haploid *S. cerevisiae*. In principle, vertebrates could reduce the probability of double fork stalls to the low levels predicted for *S. cerevisiae* by reducing the distance between licensed origins. For humans, this would mean a 500-fold reduction in inter-origin distance, and a licensed origin every ∼50 bp. This is clearly impossible, given the 70 bp footprint of a single MCM2-7 double hexamer (40,41). Estimates for the abundance of MCM2-7 on chromosomal DNA in vertebrates range from one double hexamer per 3 kb in rapidly dividing *Xenopus* embryos (9) to one double hexamer per 10–40 kb in human tissue culture cells (8,10). Similar estimates have been made for density of licensed ROs (4,11,57). This density of licensed origins suggests that for organisms with genome sizes significantly larger than *S. cerevisiae*, double fork stalls become almost inevitable genome-wide. For example, our equation suggests that in the human genome with licensed origins on average every 20 kb and an *R*-value of 0.7, then a double fork stall would occur in almost half of S phases even in the absence of replicative stresses. We therefore predict that organisms with large genomes will have evolved mechanisms for effectively dealing with the consequences of double fork stalls. One possible mechanism would be that under-replicated segments of DNA are unhooked from one another before mitosis, and the aberrant DNA structures resulting from this are repaired in the subsequent G1 phase (58). Our work suggests that this and similar pathways that can respond to replication failure are likely to be particularly important in vertebrates, which typically have much larger genomes than yeasts.

## SUPPLEMENTARY DATA

Supplementary Data are available at NAR Online.

## FUNDING

National Institutes of Health [U54 CA143682 to T.J.N.]; the Scottish University Life Science Alliance (to M.A.M. and T.J.N.); Biotechnology and Biological Sciences Research Council [BB/E023754/1, BB/G001596/1 to C.A.N.]; Cancer Research UK [C303/A7399 to J.J.B.]; and Wellcome Trust [WT083524, WT097945 and WT096598 to J.J.B.]. Funding for open access charge: Wellcome Trust grant [WT096598].

*Conflict of interest statement*. None declared.

## Supplementary Material

Supplementary Data
