# Supplementary material for: Replisome stall events have shaped the distribution of replication origins in the genomes of yeasts
Source: Nucleic Acids Res. 2013 Aug 19;41(21):9705–18. doi: 10.1093/nar/gkt728 (PMC3834809; doi:10.1093/nar/gkt728)
Supplement: Supplementary Data [file supp_41_21_9705__index.html]

Replisome stall events have shaped the distribution of replication origins in the genomes of yeasts — Replisome stall events have shaped the distribution of replication origins in the genomes of yeasts — Supplementary Data 

# Replisome stall events have shaped the distribution of replication origins in the genomes of yeasts

## Supplementary Data

files

**Files in this Data Supplement:**

- Supplementary Data - pdf file
