# Supplementary material for: Replisome stall events have shaped the distribution of replication origins in the genomes of yeasts
Source: Nucleic Acids Res. 2013 Aug 19;41(21):9705–18. doi: 10.1093/nar/gkt728 (PMC3834809; doi:10.1093/nar/gkt728)
Supplement: Supplementary Data [file supp_gkt728_nar-01388-v-2013-File008.pdf]

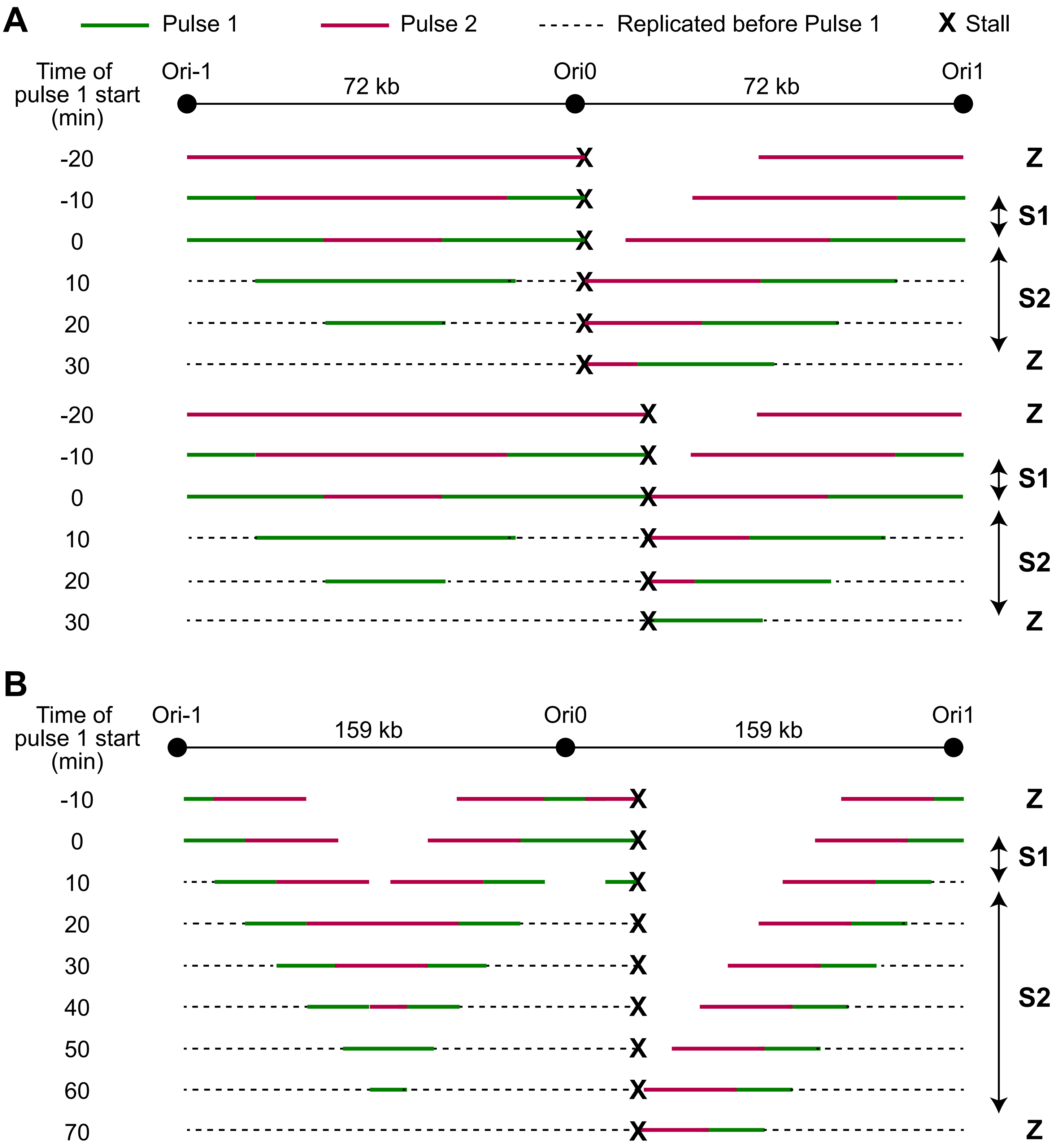

Supplementary Figure S1. Idealised representation of double label experiments. Schematic representation of experiments performed by Maya-Mendoza et al (ref 31). Cells were pulsed for 20 min with BrdU (green) then pulsed for 30 min with biotin-11-dUTP (red), and then DNA fibres were analysed. DNA replicated before the BrdU pulse is shown by a dashed line. 3 synchronously firing origins are shown. The patterns are ordered according to when the origins fired relative to the start of the BrdU pulse. 'Type 4' structures consist of a BrdU track that is adjacent to but not contiguous with a biotin track, and must result from a fork stall. They can be divided into two categories: S1, where the stall occurred during the BrdU pulse, and S2, where the stall occurred before the BrdU pulse. Z denotes patterns that would not typically fall into the 'Type 4' category (note that depending on the behaviour of the origin to the left of Ori-1, the bottom configuration in each time series might show a Type 4 pattern). The fork rate is ~1.25 kb/min. **A**. MRC5 data, mean origin-to-origin spacing 72 kb (0.5% Type 4 patterns). **B**. HeLa cell data, mean origin-to-origin spacing 159 kb (1.5% Type 4 patterns). The larger origin-to-origin spacing in HeLa cells means more stalls occur before the pulse and a correspondingly higher number of Type 4 patterns.

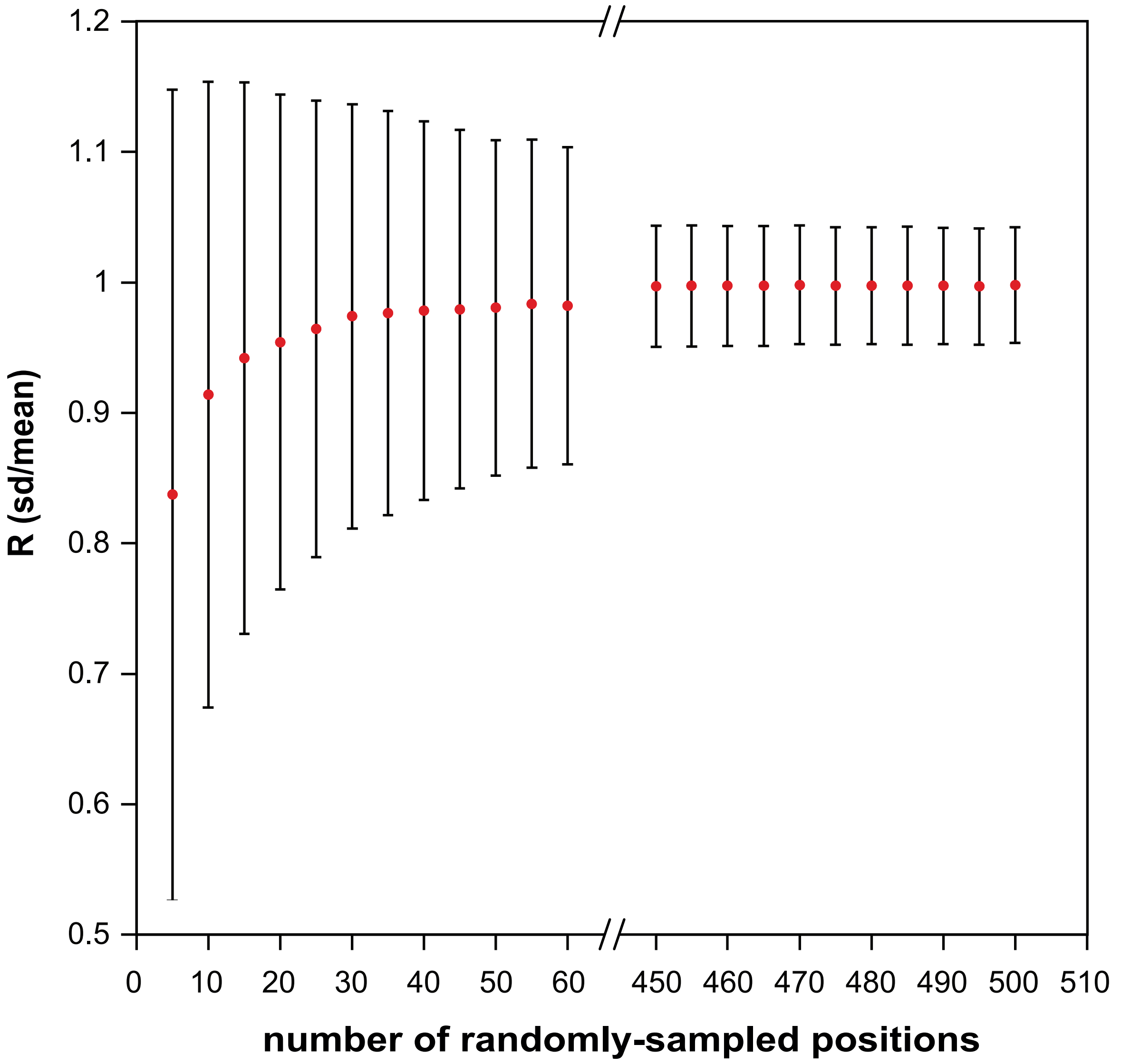

Supplementary Figure S2. *R* for randomly sampled positions on a finite line. In simulations, the number of positions indicated on the x axis were randomly selected on a finite line, and for each simulation the resulting value for *R* (the standard deviation divided by the mean of the separation between the positions) was calculated. Red dots show the mean value of *R* in10,000 iterations, and error bars show the respective standard deviation of the mean values.

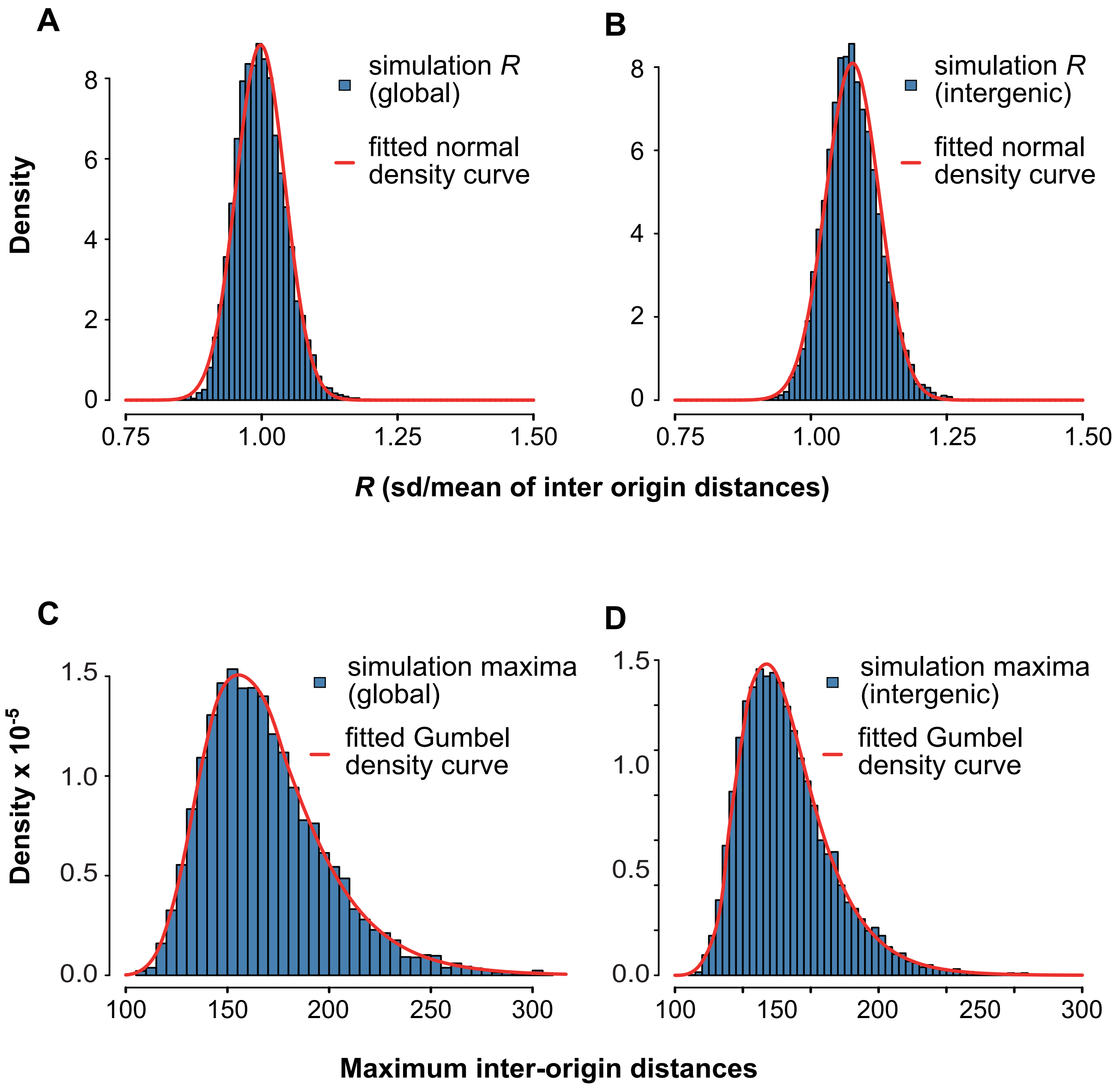

Supplementary Figure S3. Fitted distributions used for p value estimation. Histograms show the probability distribution of randomly-positioned *S. cerevisiae* origins (blue bars) compared with fitted density curves.

A, *R* values of globally distributed origins, fitted to a normal density curve.

B, *R* values of origins restricted to intergenic regions, fitted to a normal density curve.

C, Maximum inter-origin distances of globally distributed origins, fitted to a Gumbel (extreme value) density curve.

D, Maximum inter-origin distances of origins restricted to intergenic regions, fitted to a Gumbel (extreme value) density curve.

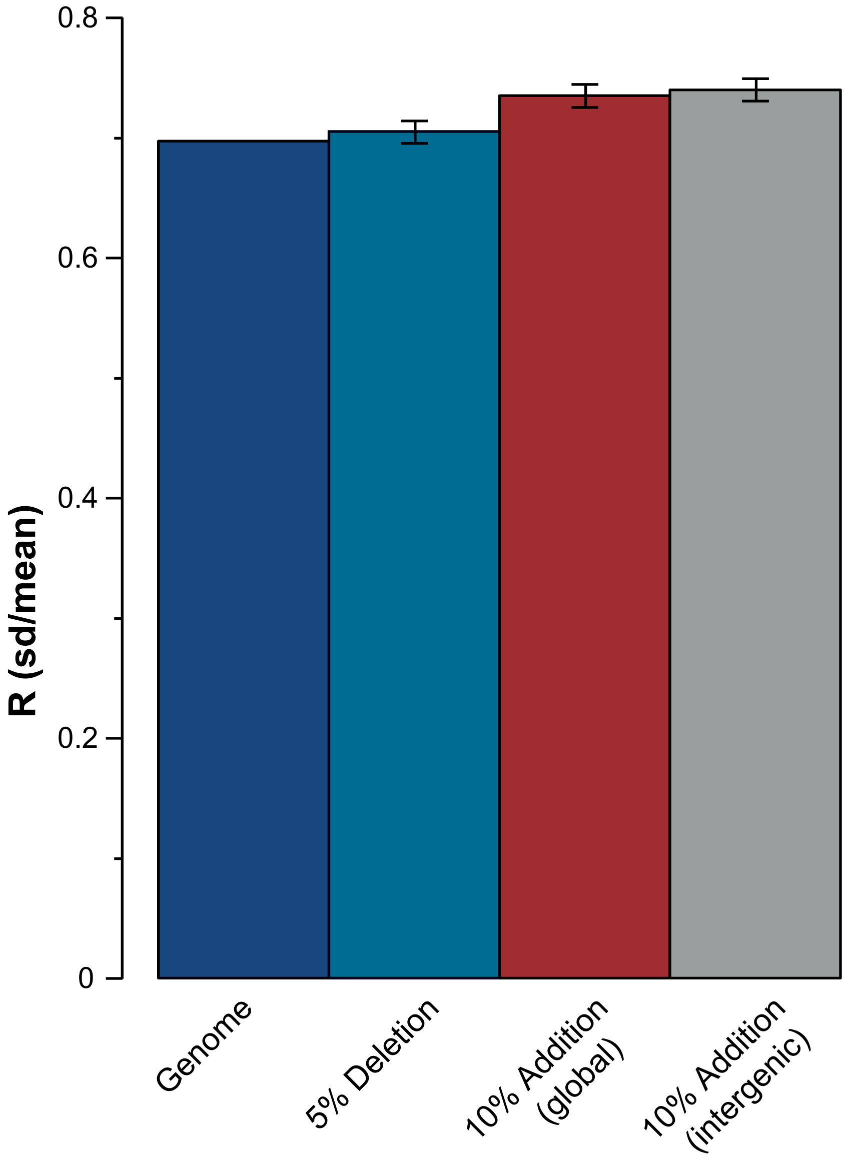

Supplementary Figure S4. Robustness of *S. cerevisiae R* value.
The robustness of the inter-origin distance *R* value (standard deviation to mean ratio) was tested by randomly deleting or adding origins in the data set. Data is shown for the deletion of 5% of the origins, or addition of an extra 10% origins either globally throughout the genome or restricted to intergenic regions. For each condition, mean and standard deviation of 10,000 simulations is shown.

A

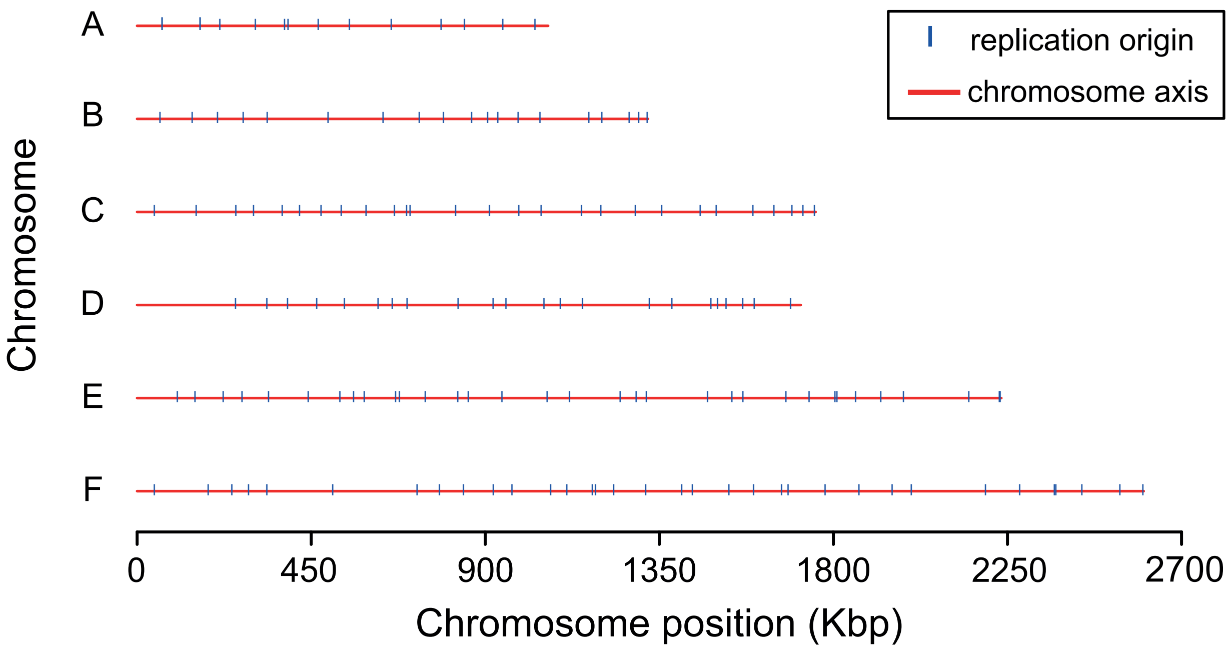
Chromosome
A
B
C
D
E
F
replication origin
chromosome axis
0
450
900
1350
1800
2250
2700
Chromosome position (Kbp)

B

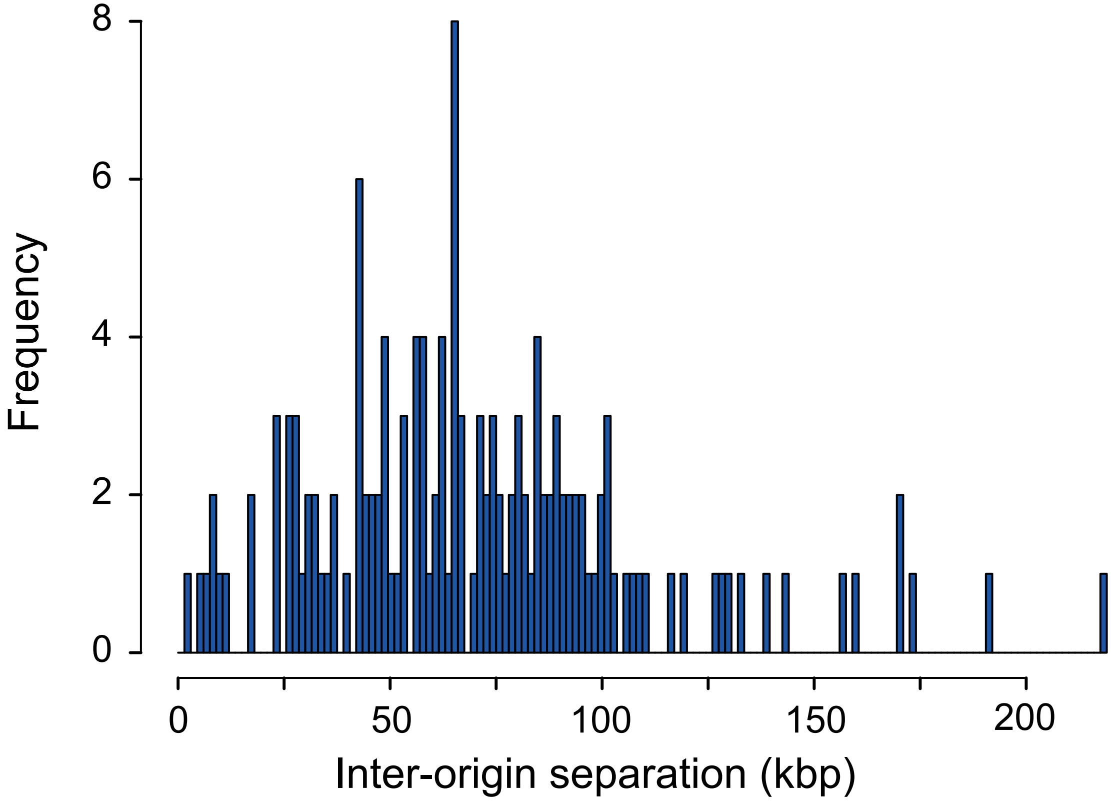
Frequency
0
2
4
6
8
0
50
100
150
200
Inter-origin separation (kbp)

C

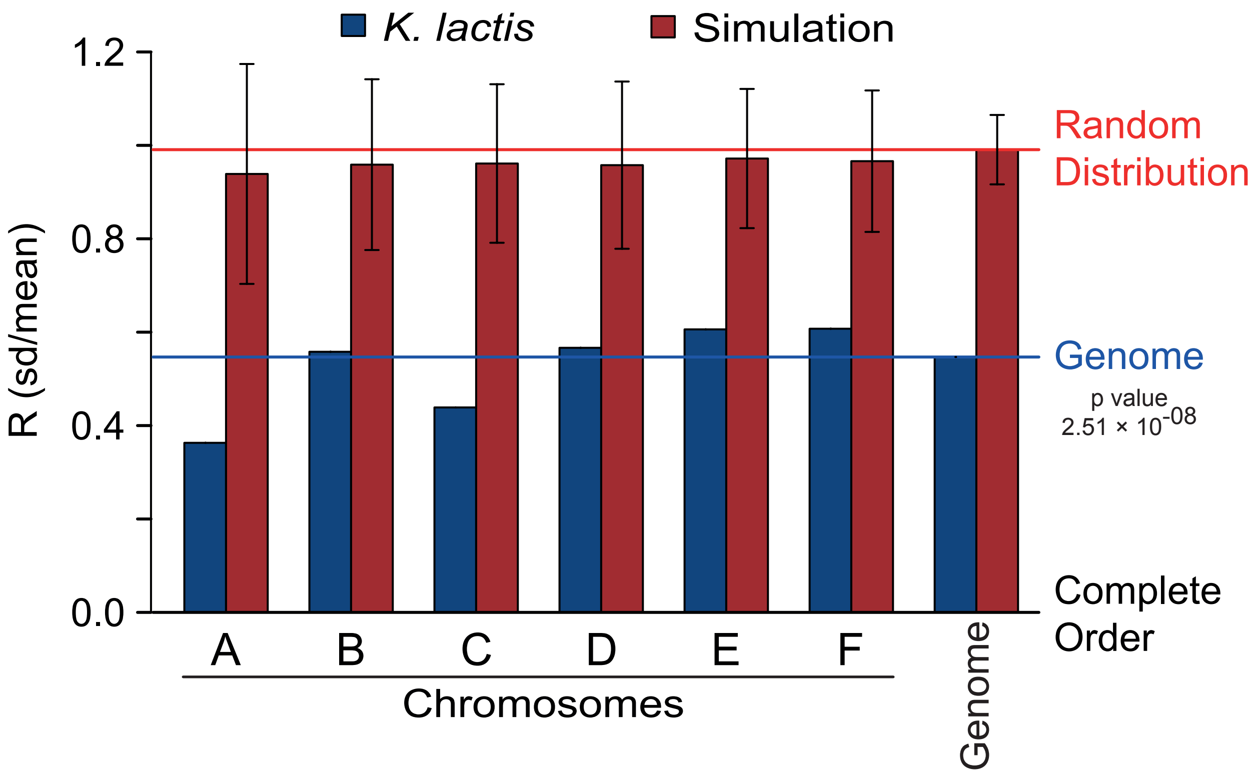
K. lactis
Simulation
1.2
0.8
0.4
0.0
R (sd/mean)
Random Distribution
Genome
p value
2.51 × 10-08
Complete Order
A
B
C
D
E
F
Genome
Chromosomes

D

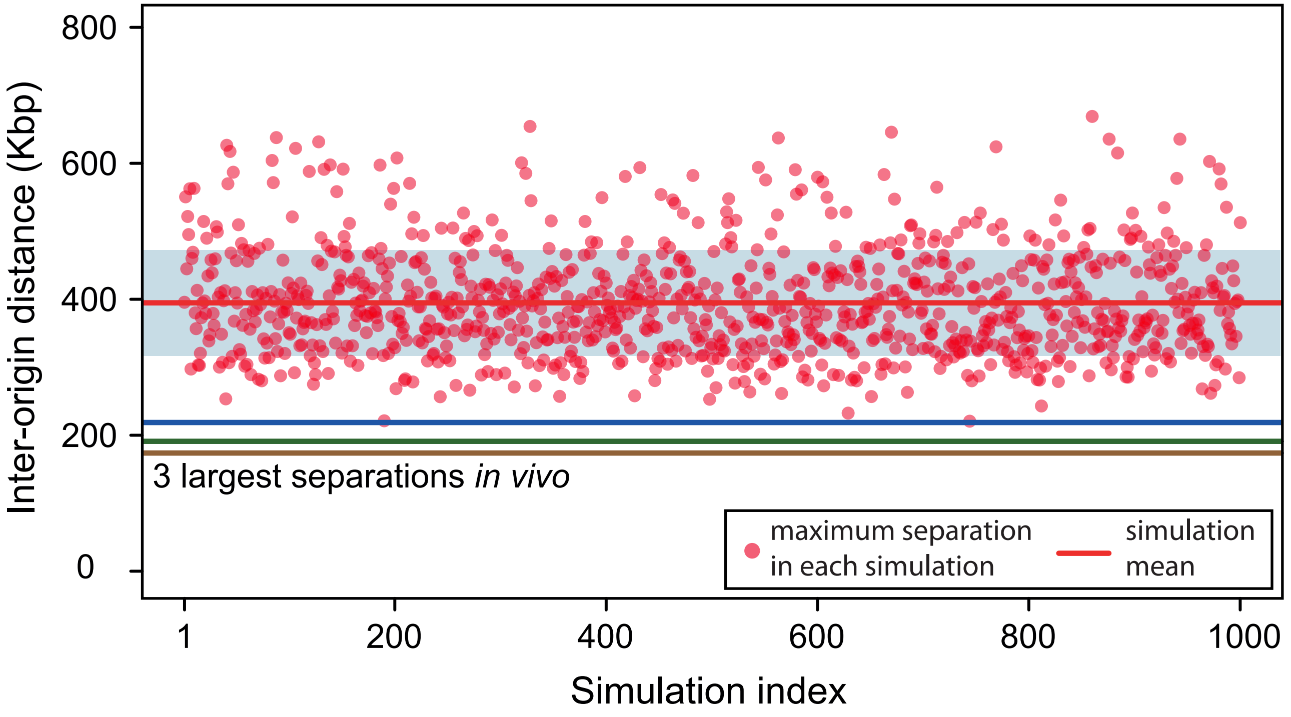
800
600
400
200
0
Inter-origin distance (Kbp)
3 largest separations in vivo
maximum separation in each simulation
simulation mean
1
200
400
600
800
1000
Simulation index

Supplementary Figure S5. Origin distribution in *Kluyveromyces lactis*.
A. Positions of 148 replication origins in all 6 *K. lactis* chromosomes.
B. Frequency histogram of inter replication origin separations (bin size 1.5 kb). The mean of the genome-wide inter-origin separation is 70.8 kbp.
C. *R* (standard deviation/mean of inter origin separations) in the genome (blue bars). Mean and standard deviation for *R* from 10,000 iterations of random sampling of positions equal to the number of replication origins in each chromosome (red bars). p value was estimated using a fitted normal distribution.
D. The three largest inter-origin distances in the genome (219, 191 and 174 kbp) are shown as horizontal lines (blue, green, and brown). A computer simulation was performed for an equal number of randomly positioned origins. Red dots are the maximum origin separation in each simulation (1000 shown). The mean of these simulated values is 399 kbp (red line) with a standard deviation of 85 (shaded box around the red line). The mean and standard deviation is calculated from 10,000 iterations in the simulation. The genomic maximum inter-origin distance (219 kb) had a p value of 2.37×10^{-4} against the simulation (from a fitted Gumbel distribution).

A

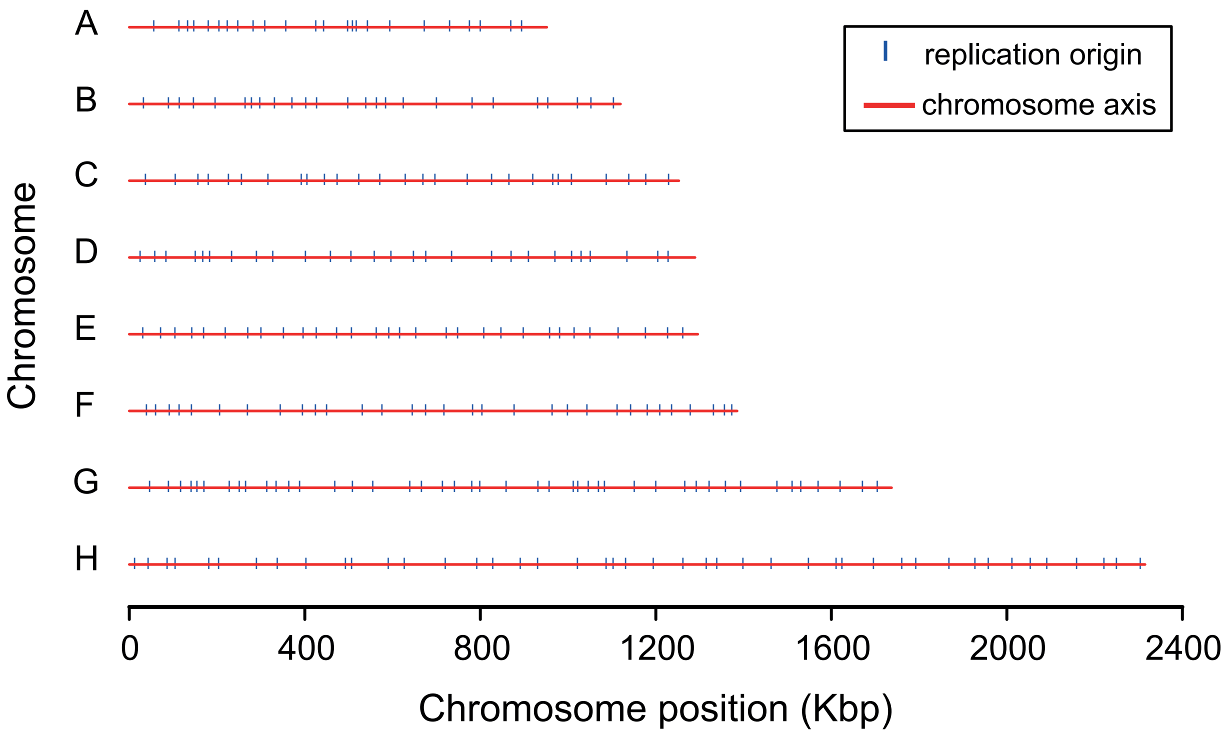
A
B
C
D
E
F
G
H
Chromosome
replication origin
chromosome axis
0
400
800
1200
1600
2000
2400
Chromosome position (Kbp)

B

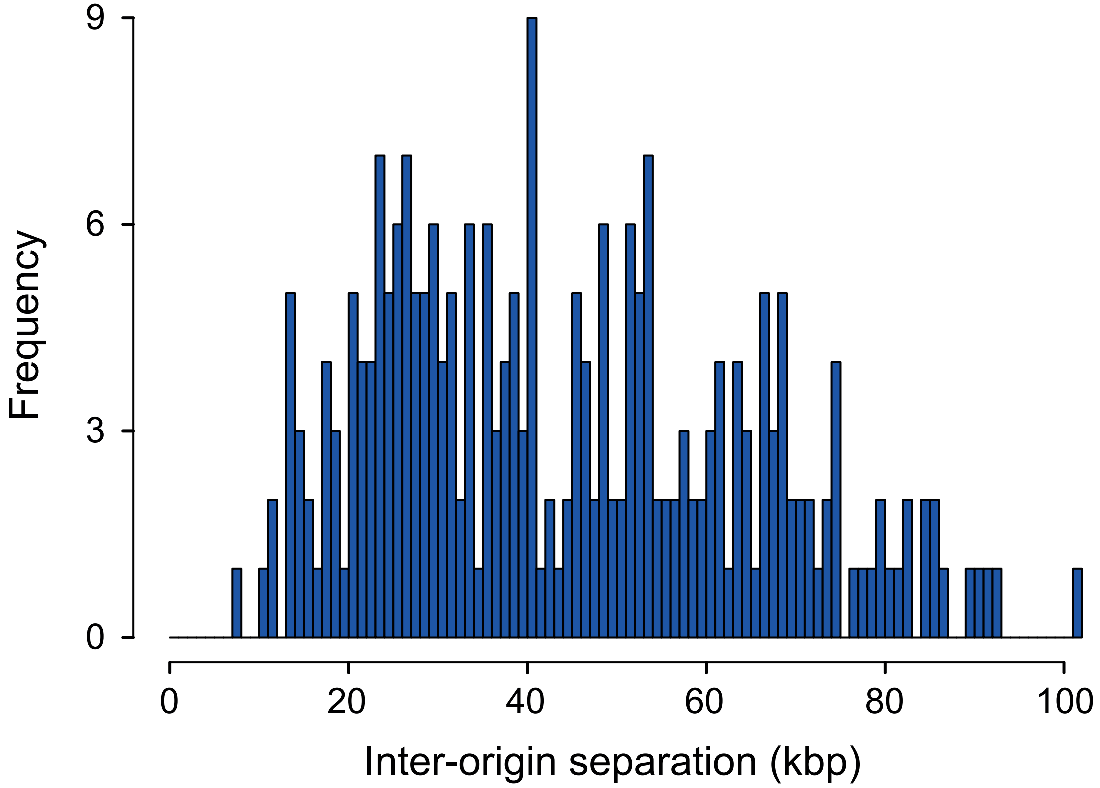
9
6
3
0
Frequency
0
20
40
60
80
100
Inter-origin separation (kbp)

C

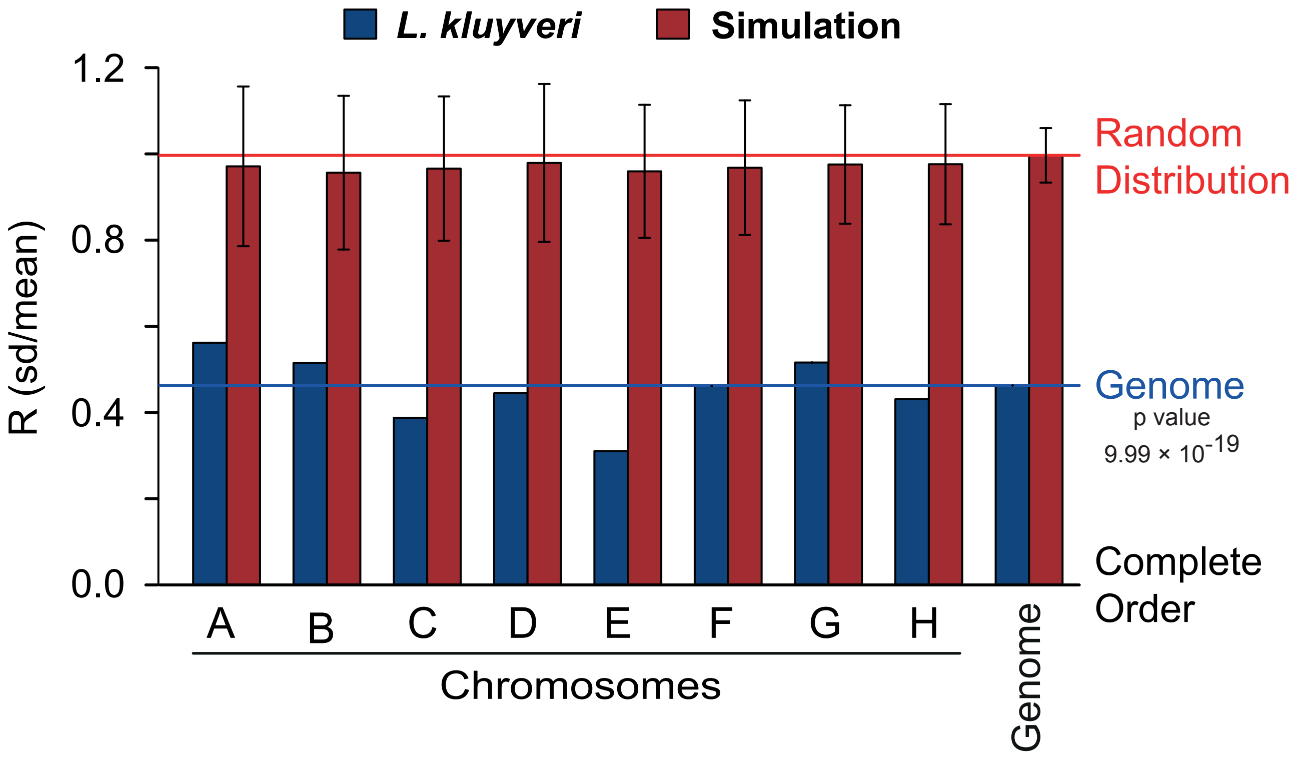
L. kluyveri
Simulation
R (sd/mean)
1.2
0.8
0.4
0.0
Random Distribution
Genome
p value
9.99 × 10-19
Complete Order
A
B
C
D
E
F
G
H
Genome
Chromosomes

D

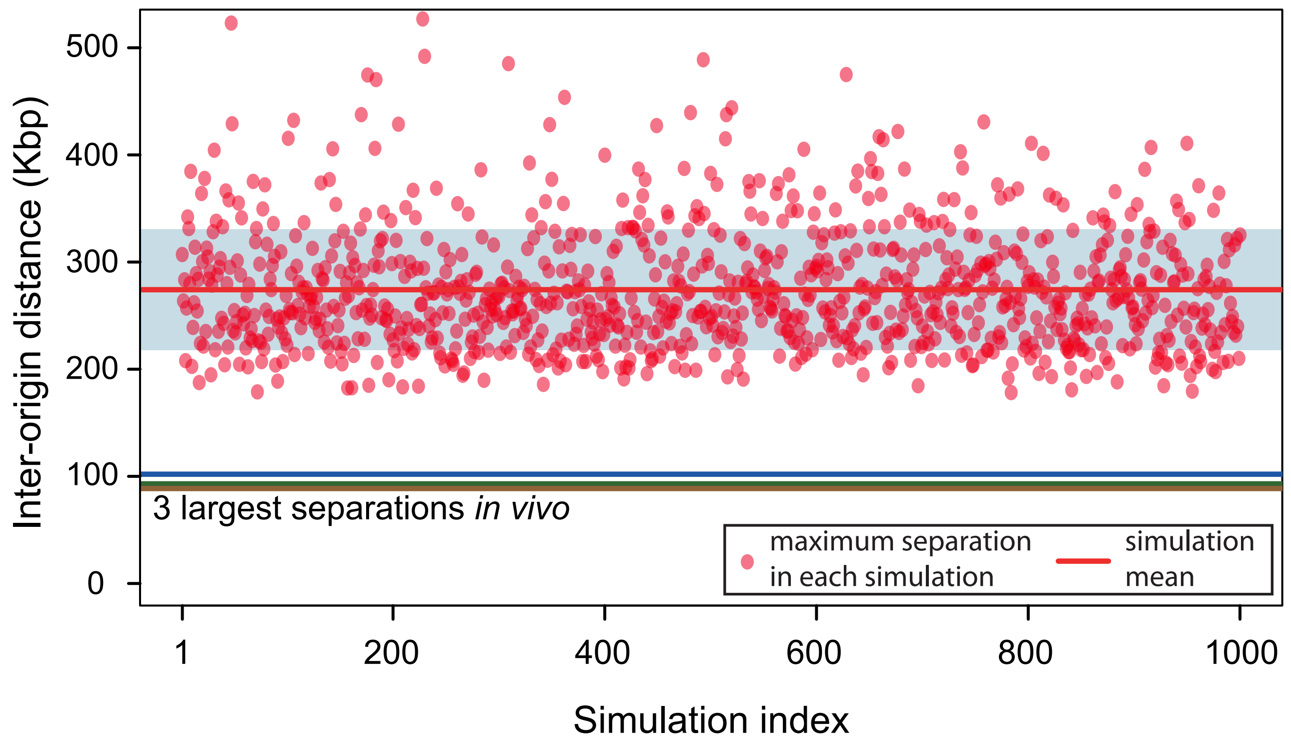
Inter-origin distance (Kbp)
500
400
300
200
100
0
3 largest separations in vivo
maximum separation in each simulation
simulation mean
1
200
400
600
800
1000
Simulation index

Supplementary Figure S6. Origin distribution in *Lachancea kluyveri*.
A. Positions of 252 replication origins in all 8 *L. kluyveri* chromosomes.
B. Frequency histogram of inter replication origin separations (bin size 1 kb). The mean of genome-wide inter origin separation is 44.34 kbp.
C. *R* (standard deviation/mean of inter origin separations) in the genome (blue bars). Mean and standard deviation for *R* from 10,000 iterations of random sampling of positions equal to the number of replication origins in each chromosome (red bars). p value was estimated from a fitted normal distribution.
D. The three largest inter-origin distances in the genome (102, 93 and 92 kbp) are shown as horizontal lines (blue, green, and brown). A computer simulation was performed for an equal number of randomly positioned origins. Red dots are the maximum origin separation in the random simulation (1000 shown). The mean of these simulated values is 273 kbp (red line) with a standard deviation of 53 (shaded box around the red line).The mean and standard deviation is calculated from 10,000 iterations in the simulation. The genomic maximum inter-origin distance (102 kb) had a p value of 8.78×10^{-16} against the simulation (from a fitted Gumbel distribution).

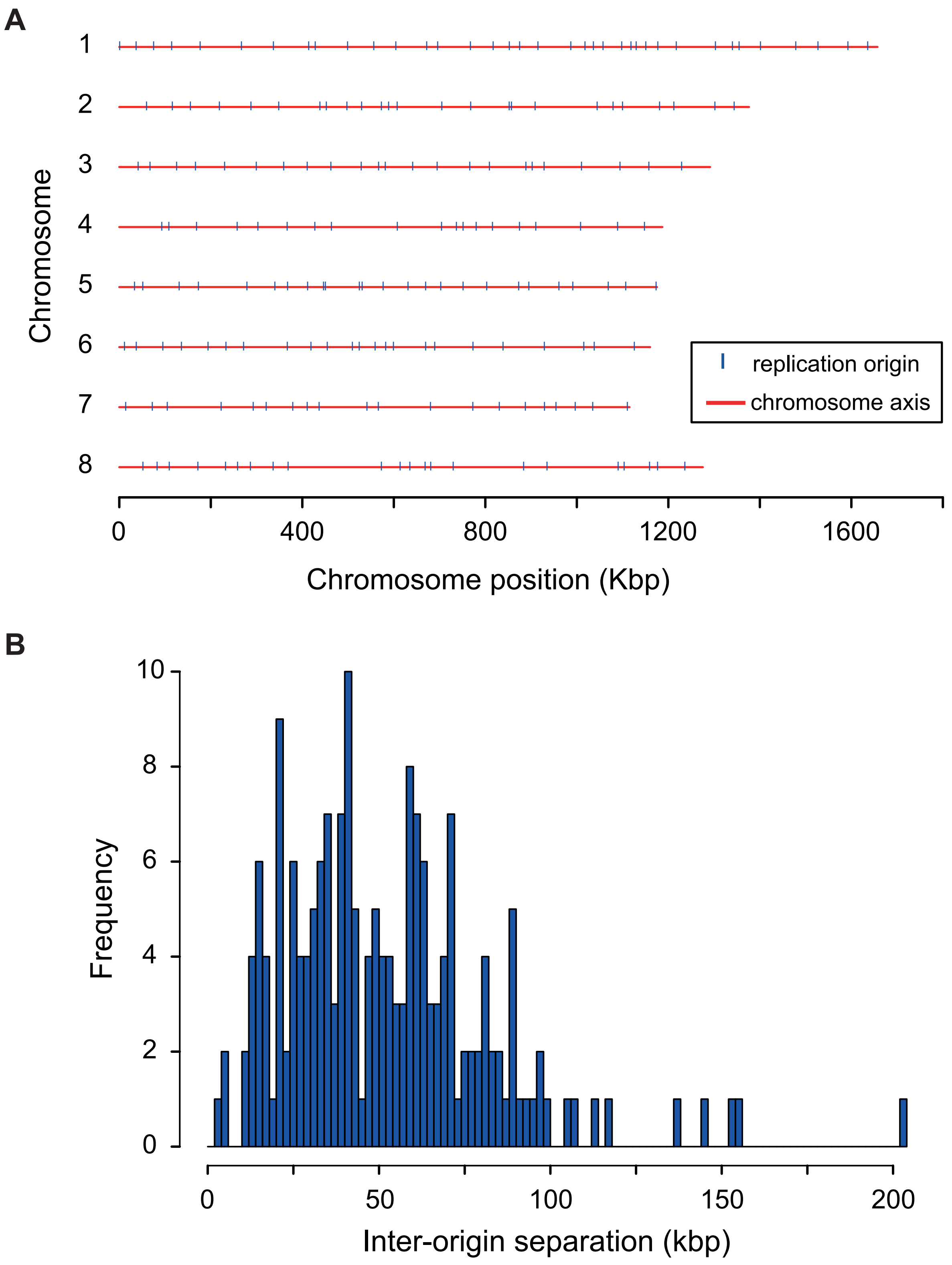
A
1
2
3
4
5
6
7
8
Chromosome
replication origin
chromosome axis
0
400
800
1200
1600
Chromosome position (Kbp)
B
10
8
6
4
2
0
Frequency
0
50
100
150
200
Inter-origin separation (kbp)

C

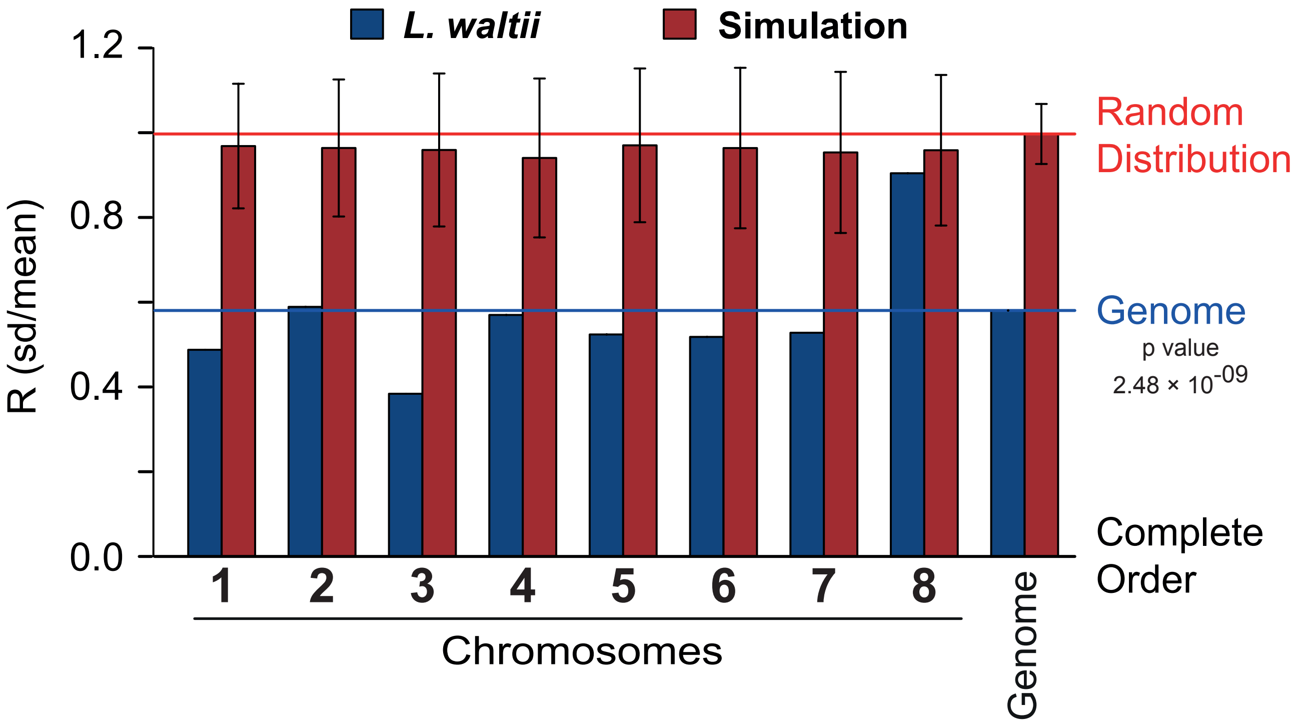
L. waltii
Simulation
1.2
0.8
0.4
0.0
R (sd/mean)
Random Distribution
Genome
p value
2.48 × 10-09
Complete Order
1
2
3
4
5
6
7
8
Chromosomes
Genome

D

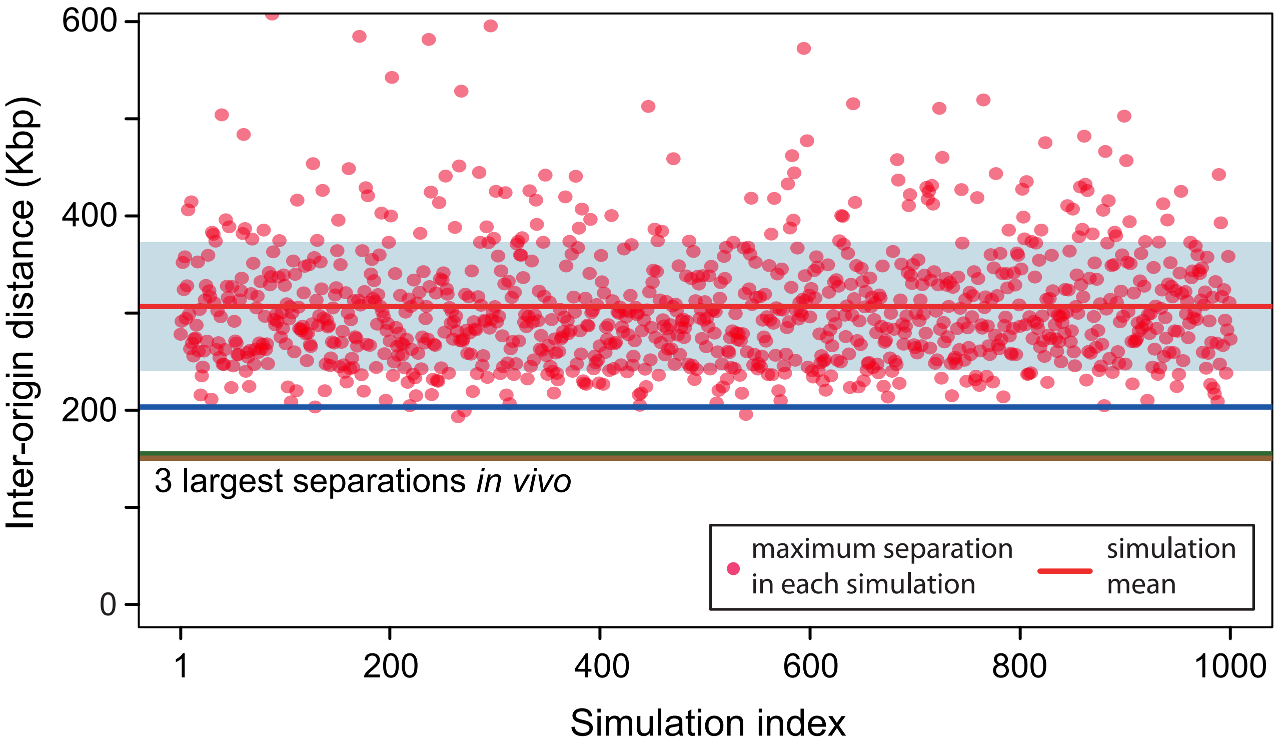
600
400
200
0
Inter-origin distance (Kbp)
3 largest separations in vivo
maximum separation in each simulation
simulation mean
1
200
400
600
800
1000
Simulation index

Supplementary Figure S7. Origin distribution in *Lachancea waltii*.
A. Positions of 194 replication origins in all 8 *L. waltii* chromosomes.
B. Frequency histogram of inter replication origin separations (bin size 2 kb). The mean of genome-wide inter origin separation is 52.13 kbp.
C. R (standard deviation/mean of inter origin separations) in the genome (blue bars). Mean and standard deviation for R from 10,000 iterations of random sampling of positions equal to the number of replication origins in each chromosome (red bars). p value was estimated from a fitted normal distribution.
D. The three largest inter-origin distances in the genome (203, 155 and 153 kbp) are shown as horizontal lines (blue, green, and brown). A computer simulation was performed for an equal number of randomly positioned origins. Red dots are the maximum origin separation in the random simulation (1,000 shown). The mean of these simulated values is 307 kbp (red line) with a standard deviation of 64 (shaded box around the red line). The mean and standard deviation is calculated from 10,000 iterations in the simulation. The genomic maximum inter-origin distance (203 kb) had a p value of 1.08×10^{-2} against the simulation (from a fitted Gumbel distribution).

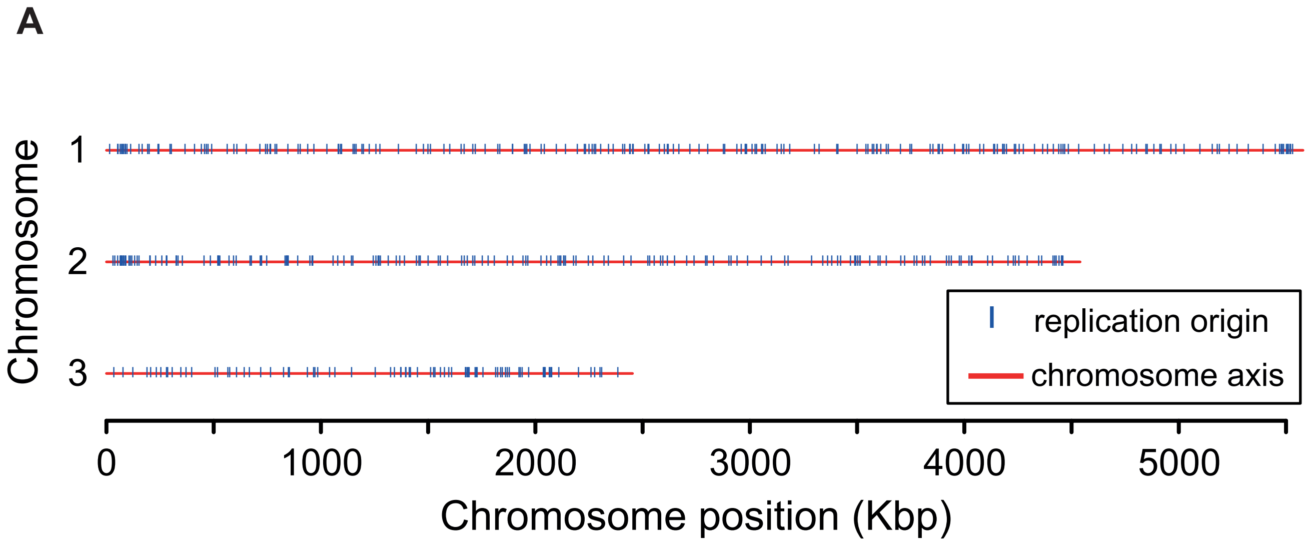
A
Chromosome
1
2
3
replication origin
chromosome axis
0
1000
2000
3000
4000
5000
Chromosome position (Kbp)

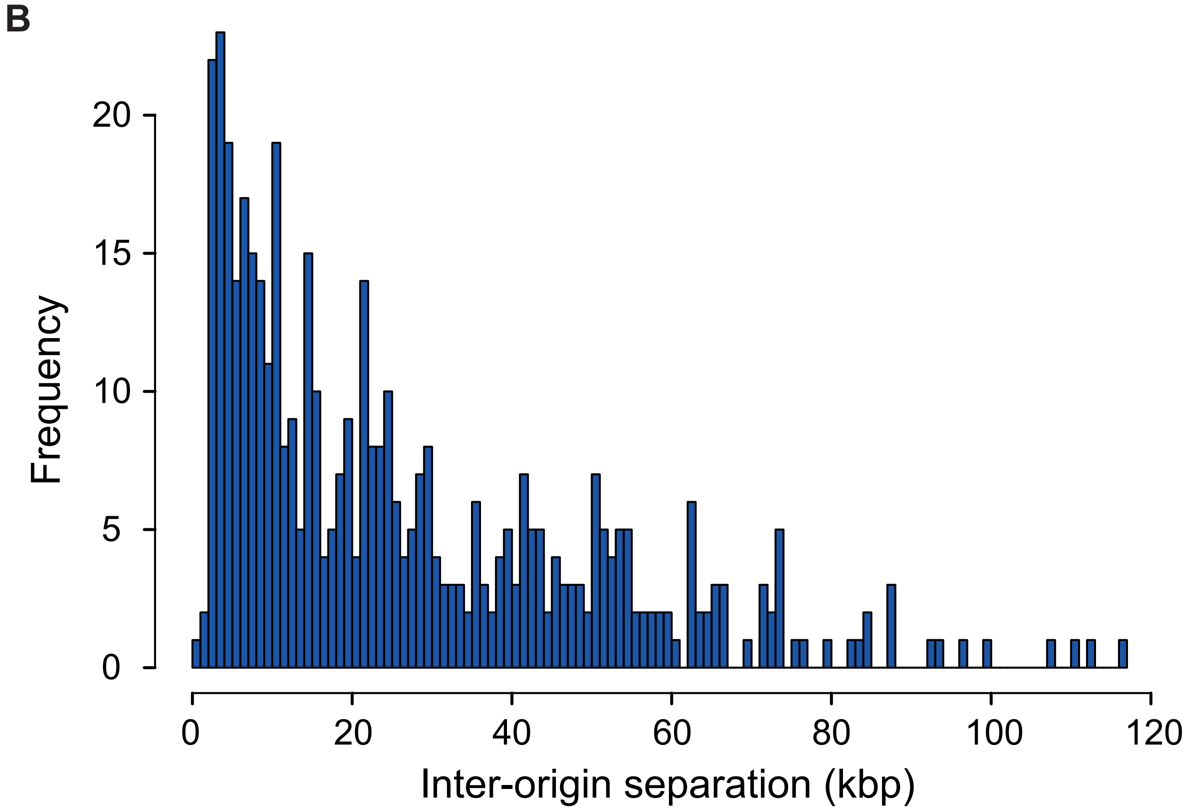
B
Frequency
0
5
10
15
20
0
20
40
60
80
100
120
Inter-origin separation (kbp)

C

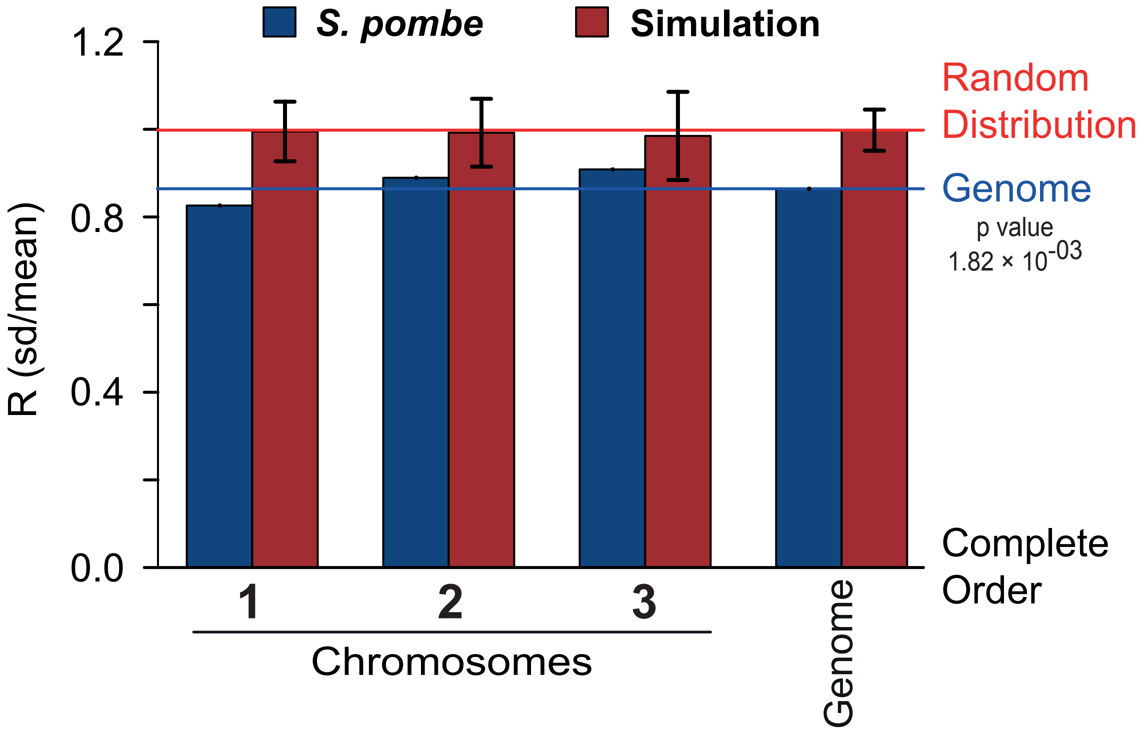

S. pombe
Simulation
1.2
0.8
0.4
0.0
R (sd/mean)
Random Distribution
Genome
p value
1.82 × 10-03
Complete Order
1
2
3
Chromosomes
Genome

D

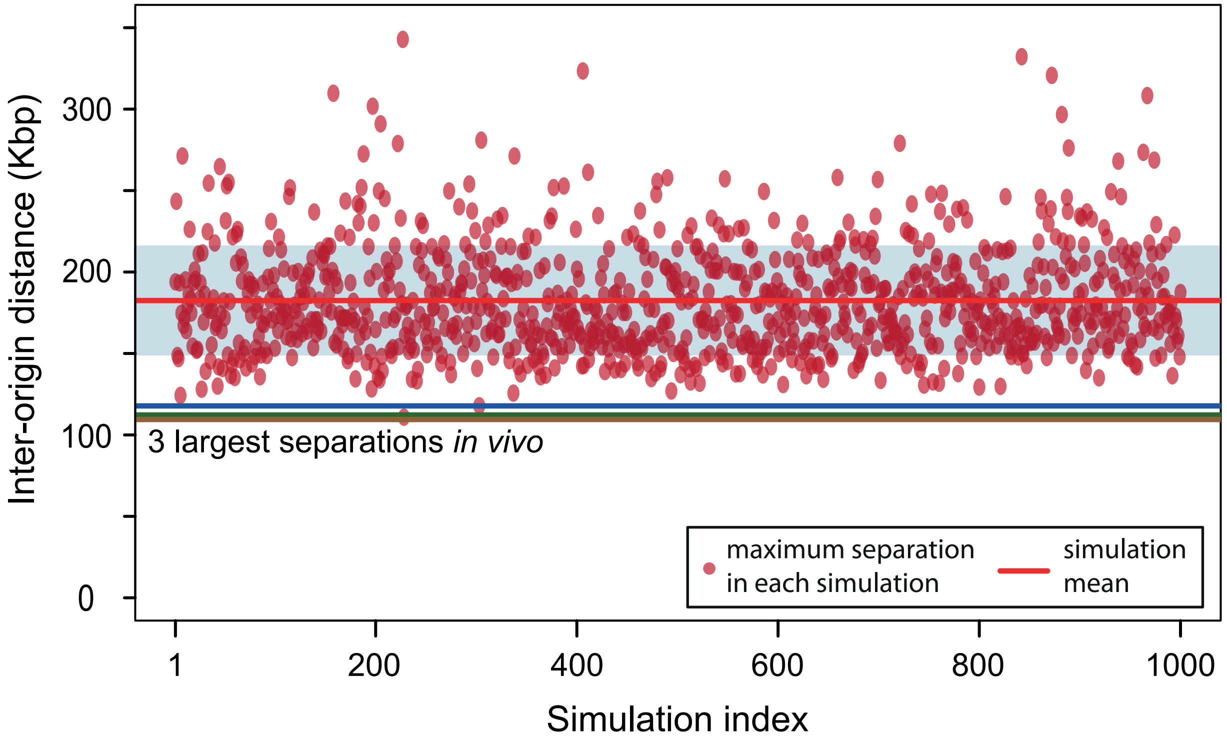

300
200
100
0
Inter-origin distance (Kbp)
3 largest separations in vivo
maximum separation in each simulation
simulation mean
1
200
400
600
800
1000
Simulation index

Supplementary Figure S8. Origin distribution in *Schizosaccharomyces pombe*.
A. Positions of 460 replication origins in all 3 *S. pombe* chromosomes.
B. Frequency histogram of inter replication origin separations (bin size 1 kb). The mean of genome-wide inter origin separation is 26.9 kb.
C. *R* (standard deviation/mean of inter origin separations) in the genome (blue bars). Mean and standard deviation for R from 10,000 iterations of random sampling of positions equal to the number of replication origins in each chromosome (red bars). p value was estimated from a fitted normal distribution.
D. The three largest inter-origin distances in the genome (116, 112 and 111 kb) are shown as horizontal lines (blue, green, and brown). A computer simulation was performed for an equal number of randomly positioned origins. Red dots are the maximum origin separation in the random simulation (1,000 shown). The mean of these simulated values is 183 kbp (red line) with a standard deviation of 34 (shaded box around the red line). The mean and standard deviation was calculated from 10,000 iterations in the simulation. The genomic maximum inter-origin distance (116 kb) had a p value of 9.52×10^{-4} against the simulation (from a fitted Gumbel distribution).

Supplementary Dataset 1: *Saccharomyces cerevisiae* ARS list

Chromosome	Start position	End position	ARS name
chr1	650	1791	ARS102
chr1	6136	7136	ARS102.5
chr1	7998	8548	ARS103
chr1	30946	31184	ARS104
chr1	40716	43300	ARS105
chr1	70258	70491	ARS106
chr1	124350	124599	ARS107
chr1	136900	137900	ARS107.5
chr1	146703	147690	ARS108
chr1	159906	160127	ARS109
chr1	176154	176402	ARS110
chr1	214879	215635	ARS111
chr1	222871	224037	ARS112
chr1	226100	227000	ARS113
chr2	39	686	ARS200
chr2	6123	7127	ARS201
chr2	28933	29152	ARS201.5
chr2	38623	39068	ARS201.7
chr2	63186	63421	ARS202
chr2	93410	93811	ARS203
chr2	142868	144016	ARS206
chr2	170049	170298	ARS207
chr2	177529	177877	ARS207.1
chr2	198193	198434	ARS207.5
chr2	209187	210063	ARS207.8
chr2	237644	237879	ARS208
chr2	254890	255136	ARS209
chr2	283015	283913	ARS210.5
chr2	326099	326335	ARS211
chr2	378434	379194	ARS212
chr2	389245	390368	ARS213
chr2	407831	408064	ARS214
chr2	417739	418035	ARS215
chr2	440859	441697	ARS215.5
chr2	486661	486909	ARS216
chr2	516805	517805	ARS217
chr2	539137	539699	ARS218
chr2	591424	591713	ARS219
chr2	611269	613200	ARS219.5
chr2	622625	622894	ARS220
chr2	631934	632246	ARS221
chr2	675947	676667	ARS221.5
chr2	704250	704521	ARS222
chr2	707158	708262	ARS222.5
chr2	720601	721038	ARS223
chr2	741512	741802	ARS224
chr2	757390	757621	ARS225
chr2	773918	774348	ARS227

chr2	792116	792340	ARS228
chr2	796259	797162	ARS228.5
chr2	801930	802617	ARS229
chr3	837	1551	ARS300
chr3	11145	11401	ARS301
chr3	11303	14854	ARS302
chr3	14574	14849	ARS302
chr3	14870	15213	ARS303
chr3	15213	16274	ARS320
chr3	30199	30657	ARS304
chr3	39158	39706	ARS305
chr3	74457	74677	ARS306
chr3	108775	109291	ARS307
chr3	114314	114933	ARS308
chr3	131978	132322	ARS309
chr3	166494	167340	ARS310
chr3	194256	194505	ARS313
chr3	197369	197601	ARS314
chr3	203876	207534	ARS314.5
chr3	224807	225053	ARS315
chr3	272844	273088	ARS316
chr3	292524	292826	ARS317
chr3	294396	295027	ARS318
chr3	315346	316232	ARS319
chr4	137	1392	ARS400
chr4	15492	15739	ARS403
chr4	21265	22262	ARS402.5
chr4	46181	46237	ARS404
chr4	85945	86177	ARS405
chr4	100380	101355	ARS405.5
chr4	123617	123902	ARS406
chr4	137445	137808	ARS407
chr4	157744	158235	ARS408
chr4	212420	212669	ARS409
chr4	232140	232618	ARS409.3
chr4	235935	236184	ARS409.5
chr4	253789	254038	ARS410
chr4	316719	317111	ARS412
chr4	329564	329813	ARS413
chr4	350835	351804	ARS413.5
chr4	408070	408312	ARS414
chr4	435056	435388	ARS415
chr4	462430	462700	ARS416
chr4	483846	484091	ARS417
chr4	505336	505578	ARS417.5
chr4	555224	555461	ARS418
chr4	567490	567737	ARS419
chr4	629072	629669	ARS420
chr4	639859	640108	ARS421
chr4	702879	703125	ARS422
chr4	748384	748630	ARS422.5
chr4	753159	753391	ARS423

chr4	806044	806270	ARS425
chr4	844597	845593	ARS426.5
chr4	903824	904655	ARS427.5
chr4	913780	914029	ARS428
chr4	921682	921930	ARS429
chr4	942983	943975	ARS429.5
chr4	1016624	1016922	ARS430
chr4	1033069	1034182	ARS430.5
chr4	1057828	1058076	ARS431
chr4	1109955	1110196	ARS431.5
chr4	1130672	1131155	ARS431.7
chr4	1159250	1159499	ARS432
chr4	1165998	1166221	ARS432.5
chr4	1240869	1241098	ARS433
chr4	1276212	1276440	ARS434
chr4	1302579	1302819	ARS435
chr4	1353494	1353667	ARS437
chr4	1385151	1385630	ARS439.5
chr4	1404277	1404512	ARS440
chr4	1447298	1448928	ARS441
chr4	1461849	1462161	ARS442
chr4	1486905	1487149	ARS446
chr4	1502624	1503221	ARS447
chr5	4097	4185	ARS502
chr5	6464	7230	ARS503
chr5	8786	10019	ARS504
chr5	11713	12194	ARS504.2
chr5	59282	59516	ARS507
chr5	93977	94218	ARS508
chr5	145539	145782	ARS510
chr5	173636	173874	ARS511
chr5	192493	192926	ARS511.5
chr5	212381	212630	ARS512
chr5	276127	276526	ARS513.5
chr5	278073	278355	ARS513.7
chr5	287504	287750	ARS514
chr5	301565	302061	ARS514.5
chr5	353504	353751	ARS516
chr5	406747	406949	ARS517
chr5	438929	439178	ARS518
chr5	442412	442731	ARS519
chr5	498417	499343	ARS520
chr5	520767	521025	ARS521
chr5	549560	549809	ARS522
chr5	569020	570085	ARS523
chr6	5435	6155	ARS600.1
chr6	12174	13028	ARS600.2
chr6	18682	19864	ARS600.3
chr6	19669	20826	ARS600.4
chr6	32472	32995	ARS601
chr6	32666	33247	ARS602
chr6	68690	68869	ARS603

chr6	104456	104695	ARS603.1
chr6	118631	118952	ARS603.5
chr6	127745	128066	ARS604
chr6	135979	136080	ARS605
chr6	167606	168041	ARS606
chr6	199382	199493	ARS607
chr6	216344	216692	ARS608
chr6	253316	253800	ARS608.5
chr6	256263	256418	ARS609
chr6	269404	270022	ARS610
chr7	17629	18112	ARS700.5
chr7	64279	64528	ARS702
chr7	112080	112319	ARS704
chr7	117471	117955	ARS706
chr7	163180	163447	ARS707
chr7	187241	187651	ARS709
chr7	203917	204159	ARS710
chr7	231909	231984	ARS711
chr7	240972	241441	ARS712
chr7	285951	286246	ARS714
chr7	352695	352917	ARS716
chr7	388658	388892	ARS717
chr7	421093	421342	ARS718
chr7	484932	485160	ARS719
chr7	508729	508978	ARS720
chr7	568490	568738	ARS721
chr7	574622	574916	ARS722
chr7	607176	607619	ARS724.5
chr7	653611	654091	ARS726
chr7	659809	660054	ARS727
chr7	715273	715556	ARS728
chr7	777967	778216	ARS729
chr7	834492	834736	ARS731
chr7	888350	888599	ARS731.5
chr7	916675	917170	ARS732
chr7	977730	977979	ARS733
chr7	999448	999695	ARS734
chr7	1002182	1002571	ARS735
chr7	1063254	1063715	ARS735.5
chr7	1082959	1084336	ARS131a
chr8	5158	6168	ARS131n
chr8	7534	7782	ARS802
chr8	45589	46076	ARS804
chr8	64255	64489	ARS805
chr8	94759	95192	ARS805.5
chr8	111293	111766	ARS805.7
chr8	133347	133591	ARS807
chr8	140340	141265	ARS808
chr8	168531	168773	ARS809
chr8	213179	213861	ARS810
chr8	245719	245968	ARS813
chr8	296882	297475	ARS815

chr8	359469	359928	ARS816
chr8	392148	392391	ARS818
chr8	406012	406508	ARS818.5
chr8	447619	447853	ARS820
chr8	474309	474793	ARS821
chr8	501751	501992	ARS822
chr8	520016	520630	ARS823
chr8	535403	535836	ARS823.5
chr8	555996	556327	ARS824
chr9	33335	33803	ARS905
chr9	73803	74220	ARS907
chr9	80058	80557	ARS908
chr9	105821	106048	ARS909
chr9	136094	136335	ARS911
chr9	162936	163302	ARS911.5
chr9	175034	175355	ARS912
chr9	214675	214826	ARS913
chr9	245694	245932	ARS913.5
chr9	247579	247800	ARS914
chr9	310583	311070	ARS916
chr9	341853	342096	ARS919
chr9	357156	357393	ARS920
chr9	411817	412053	ARS922
chr9	432496	432994	ARS922.5
chr10	64	577	ARS1001
chr10	7444	7943	ARS1002
chr10	16123	16762	ARS1003
chr10	23661	24158	ARS1004
chr10	67467	67949	ARS1005
chr10	99359	99796	ARS1006
chr10	113226	113828	ARS1007
chr10	161435	161860	ARS1007.5
chr10	203729	204614	ARS1008
chr10	228248	228740	ARS1009
chr10	248600	249300	ARS1009.5
chr10	298471	298952	ARS1010
chr10	336976	337225	ARS1011
chr10	374575	374818	ARS1012
chr10	375401	375923	ARS1013
chr10	416888	417134	ARS1014
chr10	442248	442658	ARS1015
chr10	454276	455248	ARS1016
chr10	459029	459588	ARS1017
chr10	501595	502525	ARS1017.5
chr10	540239	540474	ARS1018
chr10	609000	609750	ARS1018.5
chr10	612542	612975	ARS1019
chr10	654069	654309	ARS1020
chr10	683328	683817	ARS1021
chr10	711590	711837	ARS1022
chr10	729677	730208	ARS1023
chr10	736728	736970	ARS1024

chr10	744102	744703	ARS1025
chr11	42110	42563	ARS1102.5
chr11	55670	55917	ARS1103
chr11	98329	98568	ARS1104.5
chr11	145433	145893	ARS1105.5
chr11	152934	153173	ARS1106
chr11	196038	196284	ARS1106.3
chr11	199886	200537	ARS1106.5
chr11	213080	213385	ARS1106.7
chr11	257390	257839	ARS1107
chr11	302152	302524	ARS1107.5
chr11	329322	329571	ARS1109
chr11	388607	388902	ARS1112
chr11	416822	417055	ARS1113
chr11	444561	445028	ARS1113.5
chr11	447657	447892	ARS1114
chr11	454453	459197	ARS1114.5
chr11	516653	516902	ARS1116
chr11	530070	530560	ARS1117
chr11	581468	581706	ARS1118
chr11	611874	612107	ARS1120
chr11	642355	642602	ARS1123
chr12	51860	52210	ARS1203
chr12	76711	77163	ARS1205
chr12	91417	91659	ARS1206
chr12	93166	93659	ARS1206.5
chr12	150914	151421	ARS1208
chr12	156646	156883	ARS1209
chr12	222410	222792	ARS1210
chr12	231179	231422	ARS1211
chr12	243527	243960	ARS1211.5
chr12	289220	289469	ARS1212
chr12	343577	344033	ARS1212.5
chr12	373156	373400	ARS1213
chr12	412668	412897	ARS1215
chr12	450485	450726	ARS1216
chr12	458826	459141	ARS1216.5
chr12	512868	513117	ARS1217
chr12	602938	603155	ARS1218
chr12	622672	623123	ARS1219
chr12	644937	645426	ARS1219.7
chr12	659823	660072	ARS1220
chr12	687249	687738	ARS1220.5
chr12	730275	730832	ARS1222
chr12	744942	745179	ARS1223
chr12	794020	794269	ARS1226
chr12	821907	822336	ARS1227
chr12	888569	888810	ARS1227.5
chr12	928051	928540	ARS1229
chr12	947908	948358	ARS1231
chr12	1007180	1007470	ARS1232
chr12	1013789	1014017	ARS1233

chr12	1023967	1024207	ARS1234
chr12	1059486	1059919	ARS1235
chr13	31687	31935	ARS1303
chr13	39922	40355	ARS1304
chr13	49645	50141	ARS1304.5
chr13	73874	74373	ARS1304.7
chr13	94216	94463	ARS1305
chr13	137299	137548	ARS1307
chr13	158846	159279	ARS1307.5
chr13	183793	184037	ARS1308
chr13	263062	263296	ARS1309
chr13	286782	287067	ARS1310
chr13	370976	371221	ARS1312
chr13	432851	433347	ARS1315
chr13	468177	468468	ARS1316
chr13	503346	504087	ARS1319
chr13	535595	535843	ARS1320
chr13	554392	554750	ARS1322
chr13	611273	611488	ARS1323
chr13	634479	634714	ARS1324
chr13	649307	649551	ARS1325
chr13	688766	689221	ARS1326
chr13	758222	758470	ARS1327
chr13	772629	772878	ARS1328
chr13	805116	805338	ARS1329
chr13	815341	815567	ARS1330
chr13	865288	865683	ARS1331.5
chr13	878619	879115	ARS1331.7
chr13	897804	898040	ARS1332
chr14	28467	28699	ARS1405
chr14	61597	61894	ARS1406
chr14	89528	89802	ARS1407
chr14	126488	126981	ARS1410
chr14	169566	169804	ARS1411
chr14	196055	196291	ARS1412
chr14	250259	250506	ARS1413
chr14	279875	280108	ARS1414
chr14	321917	322210	ARS1415
chr14	352509	352996	ARS1311.7
chr14	412263	412493	ARS1417
chr14	449343	449588	ARS1419
chr14	498987	499232	ARS1420
chr14	545966	546201	ARS1421
chr14	561106	561384	ARS1422
chr14	609458	609706	ARS1424
chr14	635660	635901	ARS1426
chr14	691482	691727	ARS1427
chr14	713642	714136	ARS1428
chr14	738550	738982	ARS1428.5
chr14	764001	764500	ARS1429
chr15	35667	35903	ARS1506.5
chr15	72636	72872	ARS1507

chr15	85195	85444	ARS1508
chr15	113843	114084	ARS1509
chr15	128922	130182	ARS1509.3
chr15	154972	155462	ARS1509.5
chr15	166974	167220	ARS1510
chr15	227481	228117	ARS1510.5
chr15	277529	277778	ARS1511
chr15	308969	309462	ARS1512
chr15	337279	337528	ARS1513
chr15	436732	436966	ARS1513.5
chr15	489645	490129	ARS1514
chr15	497144	497603	ARS1515.5
chr15	520541	521040	ARS1515.7
chr15	566409	566643	ARS1516
chr15	600885	600960	ARS1517
chr15	656632	656876	ARS1519
chr15	681112	681611	ARS1520
chr15	729739	729969	ARS1521
chr15	766617	766862	ARS1523
chr15	783344	783563	ARS1524
chr15	854735	855228	ARS1525
chr15	874190	874434	ARS1526
chr15	908288	908537	ARS1528
chr15	981454	981690	ARS1529
chr15	1053490	1053901	ARS1529.5
chr16	6778	8439	ARS1601
chr16	12290	13227	ARS1602
chr16	42976	43212	ARS1604
chr16	73038	73283	ARS1605
chr16	116505	116765	ARS1607
chr16	210467	211723	ARS1609
chr16	289483	289704	ARS1614
chr16	331678	332074	ARS1617
chr16	384536	384784	ARS1618
chr16	414032	416072	ARS1618.5
chr16	418132	418359	ARS1619
chr16	456557	456805	ARS1620.5
chr16	511619	511940	ARS1621
chr16	563822	564061	ARS1622
chr16	565046	565289	ARS1622.5
chr16	584037	584486	ARS1622.7
chr16	633868	634117	ARS1623
chr16	684383	684632	ARS1624
chr16	695432	695681	ARS1625
chr16	749094	749341	ARS1626
chr16	776921	777152	ARS1626.5
chr16	819153	819393	ARS1627
chr16	842646	842894	ARS1628
chr16	880854	881102	ARS1630
chr16	932976	933223	ARS1631
chr4	8605	8615	
chr4	476665	476675	

chr4	720675	720685	
chr4	898705	898715	
chr4	899245	899255	
chr4	1379755	1379765	
chr4	1525275	1525285	
chr5	18475	18485	
chr5	256185	256195	
chr5	316575	316585	
chr7	32925	32935	
chr7	1073775	1073785	
chr8	381035	381045	
chr8	550825	550835	
chr9	7835	7845	
chr9	16735	16745	
chr9	30665	30675	
chr9	190285	190295	
chr9	438825	438835	
chr10	643975	643985	
chr11	236045	236055	
chr11	462665	462675	
chr11	665465	665475	
chr12	11375	11385	
chr12	31875	31885	
chr12	139795	139805	
chr12	468045	468055	
chr12	786445	786455	
chr13	341665	341675	
chr15	11085	11095	
chr15	19115	19125	
chr15	348345	348355	
chr15	463995	464005	
chr15	617315	617325	
chr15	899395	899405	
chr15	998295	998305	
chr15	1083715	1083725	
chr16	90435	90445	
chr16	162505	162515	
chr16	190565	190575	
chr16	261525	261535	
chr16	298495	298505	
chr16	317525	317535	
chr16	427925	427935	
chr16	499545	499555	
chr16	553355	553365	
chr16	864235	864245	
chr16	942165	942175	
chr5	53	218	
chr7	694	859	
chr8	205	370	
chr9	202	367	
chr11	686	851	
chr12	204	369	

chr13	202	367	
chr14	161	326	
chr15	675	840	
chr16	70	235	
chr1	229445	229560	
chr2	812322	812437	
chr4	1531787	1531902	
chr5	576615	576730	
chr7	1090690	1090805	
chr8	562318	562433	
chr9	439076	439191	
chr11	665684	665799	
chr12	1077989	1078104	
chr13	923600	923715	
chr14	783509	783624	
chr15	1090965	1091080	
chr16	947789	947904	

This dataset contains:

- all confirmed origins
- likely origins, that have not yet been confirmed, but were found by two independent ChIP studies
- telomeric origins that have not yet been confirmed, but are predicted from sequence conservation.

List of telomeric origins:

Chromosome	End	Element	ACS	Distance from end (bp)
1	L	X	29237..29247	699
3	L	X	38940..38950	696
4	L	X	29134..29144	712
7	L	X	29257..29267	709
11	L	X	29230..29240	701
15	L	X	39191..39201	690
1	R	X	29233..29243	663
2	R	X	29232..29242	714
3	R	X	29200..29210	696
6	R	X	29609..29619	342
9	R	X	29217..29227	709
10	R	X	29188..29198	723
11	R	X	29125..29135	661
13	R	X	29146..29156	730
14	R	X	28982..28992	719
2	L	X-Y'	29911..29921	84
5	L	X-Y'	29927..29937	68
6	L	X-Y'	not sequenced to the end	***
	L	X-Y'	29746..29756	220
9	L	X-Y'	29704..29714	217
10	L	X-Y'	29721..29731	215
13	L	X-Y'	29729..29739	217
14	L	X-Y'	29819..29829	176
16	L	X-Y'	29910..29920	85
4	R	X-Y'	29968..29978	27
5	R	X-Y'	29841..29851	154
7	R	X-Y'	29841..29851	154
8	R	X-Y'	39595..39605	221
15	R	X-Y'	29761..29771	220
16	R	X-Y'	29824..29834	171
12	L	X-Y'-Y'	29707..29717	219
12	R	X-Y'-Y'	29911..29921	84

***at Chromosome 6 left end the sequence stops some distance (several kb) short of the end; however, this end does have a Y' element. So, the origin should be < ~220 bp from the end.
